# Effects of Feeding Bt MON810 Maize to Pigs for 110 Days on Peripheral Immune Response and Digestive Fate of the *cry1Ab* Gene and Truncated Bt Toxin

**DOI:** 10.1371/journal.pone.0036141

**Published:** 2012-05-04

**Authors:** Maria C. Walsh, Stefan G. Buzoianu, Mary C. Rea, Orla O’Donovan, Eva Gelencsér, Gabriella Ujhelyi, R. Paul Ross, Gillian E. Gardiner, Peadar G. Lawlor

**Affiliations:** 1 Teagasc, Pig Development Department, Animal and Grassland Research and Innovation Centre, Moorepark, Fermoy, County Cork, Ireland; 2 Department of Chemical and Life Sciences, Waterford Institute of Technology, Waterford, Ireland; 3 Teagasc, Moorepark Food Research Centre, Fermoy, County Cork, Ireland; 4 Alimentary Pharmabiotic Centre, University College Cork, County Cork, Ireland; 5 Central Food Research Institute, Budapest, Hungary; Argonne National Laboratory, United States of America

## Abstract

**Background:**

The objective of this study was to evaluate potential long-term (110 days) and age-specific effects of feeding genetically modified Bt maize on peripheral immune response in pigs and to determine the digestive fate of the *cry1Ab* gene and truncated Bt toxin.

**Methodology/Principal Findings:**

Forty day old pigs (n = 40) were fed one of the following treatments: 1) isogenic maize-based diet for 110 days (**isogenic**); 2) Bt maize-based diet (MON810) for 110 days (**Bt**); 3) Isogenic maize-based diet for 30 days followed by Bt maize-based diet for 80 days (**isogenic/Bt**); and 4) Bt maize-based diet (MON810) for 30 days followed by isogenic maize-based diet for 80 days (**Bt/isogenic**). Blood samples were collected during the study for haematological analysis, measurement of cytokine and Cry1Ab-specific antibody production, immune cell phenotyping and *cry1Ab* gene and truncated Bt toxin detection. Pigs were sacrificed on day 110 and digesta and organ samples were taken for detection of the *cry1Ab* gene and the truncated Bt toxin. On day 100, lymphocyte counts were higher (*P*<0.05) in pigs fed Bt/isogenic than pigs fed Bt or isogenic. Erythrocyte counts on day 100 were lower in pigs fed Bt or isogenic/Bt than pigs fed Bt/isogenic (*P*<0.05). Neither the truncated Bt toxin nor the *cry1Ab* gene were detected in the organs or blood of pigs fed Bt maize. The *cry1Ab* gene was detected in stomach digesta and at low frequency in the ileum but not in the distal gastrointestinal tract (GIT), while the Bt toxin fragments were detected at all sites in the GIT.

**Conclusions/Significance:**

Perturbations in peripheral immune response were thought not to be age-specific and were not indicative of Th 2 type allergenic or Th 1 type inflammatory responses. There was no evidence of *cry1Ab* gene or Bt toxin translocation to organs or blood following long-term feeding.

## Introduction

The introduction of genetically modified (GM) technology to crop production almost 17 years ago offered the potential for a solution to the global food crisis brought about by a world population explosion. GM technology is the fastest adopted crop technology to date as it offers the possibility of higher agronomic productivity of more nutritious food without the use of pesticides [1]. The global area under cultivation by GM crops has increased 94-fold since 1996, reaching 160 million hectares in 2011 [1] and new GM crops are continuously being developed. Transgenic maize is the second most important GM crop after soybean, occupying 51 million hectares worldwide and accounting for 32% of the global area under cultivation by GM crops [1]. Bt maize is one of the most widely grown transgenic maize varieties. It is genetically engineered to express the truncated Cry1Ab toxin from *Bacillus thuringiensis* which confers resistance to the European Corn Borer.

The safety of GM food and feed in Europe is assessed by the European Food Safety Authority (EFSA) which recommends that 90-day studies in rodents are conducted for the detection of potential unintended effects arising from GM feed consumption [2]. However, some 90-day rodent studies may be insufficient to reveal late effects and longer term studies of greater than 90 days duration may be necessary to detect unintended effects of GM ingredient consumption [3].

Abnormalities in immune response have been documented in mice fed α-amylase inhibitor peas [4]. Age-specific peripheral immune responses to Bt MON810 maize have previously been reported in mice [5] and our group has previously documented minor changes in both the peripheral and intestinal immune response in pigs following short-term feeding of Bt maize [6]. Since the release of GM crops onto the market, concerns have been raised as to the fate of the recombinant DNA once ingested. While some animal studies have been unable to detect transgenic DNA outside the gastrointestinal tract (GIT) [6], [7], [8], [9], low concentrations have been documented in the organs of pigs [10], [11].

The objectives of this study were to determine if long-term feeding and age were important factors in the peripheral immune response in pigs fed Bt maize. Another objective was to evaluate any residual effects on peripheral immune response that may emerge in older pigs having received Bt maize in early life. The study was also designed to investigate the digestive fate of transgenic DNA and protein following long-term Bt maize consumption in an animal model that closely resembles humans.

## Materials and Methods

### Ethical Approval

The pig study complied with European Union Council Directives 91/630/EEC (outlines minimum standards for the protection of pigs) and 98/58/EC (concerns the protection of animals kept for farming purposes) and was approved by, and a license obtained from, the Irish Department of Health and Children (licence number B100/4147). Ethical approval was obtained from the Teagasc and Waterford Institute of Technology ethics committees.

### Animals and Experimental Design

Forty crossbred (Large White×Landrace) entire male pigs were weaned at ∼28 days of age and were allowed *ad libitum* access to a non-GM starter diet during a 12 day basal period (day –12 to 0). The mean body weight of pigs on day 0 of the study was ∼10.6 kg. On day 0, pigs were blocked by weight and ancestry and within block randomly assigned to one of four treatments (n = 10 pigs/treatment); 1) non-GM isogenic parent line maize-based diet (Pioneer PR34N43) fed to day 110 (isogenic); 2) GM maize-based diet (Pioneer PR34N44 event MON810) fed to day 110 (Bt); 3) Non-GM isogenic parent line maize-based diet fed for 30 days followed by the GM maize-based diet fed to day 110 (isogenic/Bt); and 4) GM maize-based diet fed for 30 days followed by the non-GM isogenic parent line maize-based diet fed to day 110 (Bt/isogenic). The duration of the study was 110 days.

### Housing and Management

From weaning to day 60 of the study, pigs were penned individually in one of three similar rooms, each containing 24 pens. The pens were fully slatted (1.2 m×0.9 m) with plastic slats (Faroex, Manitoba, Canada) and plastic dividers between pens. Water was available *ad libitum* from one nipple-in-bowl drinker (BALP, Charleville-Mezieres, Cedex, France) per pen. Feed was available *ad libitum* from a single stainless steel 30 cm wide feeder per pen (O’Donovan Engineering, Coachford, Co. Cork). Temperature was controlled by a hot air heating system and an exhaust fan drawing air from under slat level, both connected to a Stienen PCS 8400 controller (Stienen BV, Nederweert, The Netherlands). The temperature was maintained at 28 to 30°C in the first week and reduced by 2°C per week to 22°C. Pigs were transferred to one of four identical finisher rooms containing 18 individual pens per room on day 60 of the study and remained there until day 110 of the study. Pens (1.81 m×1.18 m) were fully slatted with plastic panelled partitions. Ventilation was by exhaust fans and air inlets connected to a Stienen PCS 8200 controller. Temperature was maintained at 20 to 22°C. Feed was available *ad libitum* as dry pellets from stainless steel dry feed hoppers 30 cm in length (O’Donovan Engineering, Coachford, Co. Cork). Water was available *ad libitum* from one BALP drinking bowl. For the duration of the study, dietary treatments were equally represented in each room to avoid additional variation due to environmental conditions. Pigs showing signs of ill health were treated as appropriate and all veterinary treatments were recorded.

### Maize and Diets

Seeds derived from GM Bt MON810 and non-GM parent line control maize (PR34N44 and PR34N43 respectively; Pioneer Hi-Bred, Sevilla, Spain) were grown simultaneously side by side in 2007 in Valtierra, Navarra, Spain by independent tillage farmers The GM and non-GM control maize were purchased by the authors from the tillage farmers for use in this animal study. Samples from the Bt and isogenic maize were tested for the presence of the *cry1Ab* gene, pesticide contaminants, mycotoxins and carbohydrate composition as previously described by Walsh *et al.* [12].

All diets were manufactured and analyzed for proximate analysis and amino acid concentration (Table 1) as previously described by Walsh *et al*. [12]. All diets were formulated to meet or exceed the NRC [13] requirements for pigs of given weights. The non-GM starter diet was fed to all pigs from weaning (day -12) until the beginning of the study (day 0). Both isogenic and Bt maize link diets were fed from day 0 to 30, weaner diets were fed from day 31 to 60, finisher 1 diets were fed from day 61 to 100 and finisher 2 diets were fed from day 101 to day 110. Pellet hardness and durability were determined as described by Lawlor *et al*. [14].

**Table 1:** Composition of diets (as is basis, %).

Ingredient, %	Starter(d -12–0)	Link(d 0–30)		Weaner (d 31–60)		Finisher 1 (d–100)		Finisher 2 (d 101–110)	
	Isogenic^1^	Isogenic^1^	Bt^2^	Isogenic^1^	Bt^2^	Isogenic^1^	Bt^2^	Isogenic^1^	Bt^2^
Maize (Isogenic)^1^	27.33	38.88	–	65.31	–	73.38	–	79.10	–
Maize (Bt MON810)^2^	–	–	38.88	–	65.31	–	73.38	–	79.10
Soya bean meal	24.00	25.00	25.00	28.64	28.64	22.76	22.76	17.35	17.35
Lactofeed 70^3^	25.00	20.00	20.00	–	–	–	–	–	–
Immunopro 35^4^	12.50	9.00	9.00	–	–	–	–	–	–
Fat, soya oil	8.00	4.00	4.00	2.37	2.37	0.06	0.06	–	–
Lysine HCl (78.8)	0.30	0.30	0.30	0.36	0.36	0.43	0.43	0.49	0.49
DL-Methionine	0.25	0.20	0.20	0.14	0.14	0.14	0.14	0.14	0.14
L-Threonine (98)	0.12	0.12	0.12	0.15	0.15	0.17	0.17	0.19	0.19
L-Tryptophan	0.10	0.10	0.10	0.05	0.05	0.07	0.07	0.08	0.08
Weaner premix^5^	0.30	0.30	0.30	–	–	–	–	–	–
Finisher premix^6^	–	–	–	0.10	0.10	0.10	0.10	0.10	0.10
Formaxol^7^	0.20	0.20	0.20	–	–	–	–	–	–
Mycosorb^8^	–	–	–	0.20	0.20	0.20	0.20	0.20	0.20
Salt	0.30	0.30	0.30	0.30	0.30	0.30	0.30	0.30	0.30
Dicalcium phosphate	0.50	0.50	0.50	1.19	1.19	1.13	1.13	0.90	0.90
Limestone flour	1.10	1.10	1.10	1.19	1.19	1.26	1.26	1.15	1.15
***Analyzed Chemical Composition (%)***									
Dry matter	91.30	90.40	90.50	88.60	88.80	89.30	89.50	89.20	88.80
Crude protein	20.90	21.00	20.70	17.90	17.80	17.40	17.40	16.00	16.10
Oil (Acid hydrolysis)	9.60	6.20	6.30	5.20	5.40	3.20	3.10	3.20	3.10
Crude fibre	1.70	1.80	1.60	2.10	2.20	3.00	2.40	2.60	2.60
Ash	6.30	5.60	5.80	4.90	4.80	4.80	4.60	4.00	4.10
Lysine	1.55^i^	1.50	1.56	1.29	1.31	1.36	1.37	1.15	1.16
Ca^9^	8.30	7.80	7.80	8.00	8.00	8.00	8.00	7.00	7.00
P^9^	4.08	3.63	3.63	3.20	3.20	3.00	3.00	2.50	2.50
DE MJ/kg^9^	16.33	15.38	15.38	14.50	14.50	14.00	14.00	13.99	13.99
Pellet durability (g)	−10	96.40	95.80	33.00	35.00	56.10	56.80	74.80	75.10
Pellet diameter (mm)	−10	5.06	5.05	5.15	5.19	5.18	5.14	5.11	5.15
Pellet hardness (kg)	−10	4.32	4.83	1.75	1.75	2.53	2.43	3.80	3.38

1 Isogenic: non-GM parent line maize.

2 Bt; Bt MON810 maize.

3 Lactofeed 70 contains 70% lactose, 11.5% protein, 0.5% oil, 7.5% ash and 0.5% fibre (Volac, Cambridge, UK).

4 Immunopro 35 contains whey protein powder - protein 35% (Volac, Cambridge, UK).

5 Premix provided per kg of complete diet: Cu, 155 mg; Fe, 90 mg; Mn, 47 mg; Zn, 120 mg, I, 0.6 mg; Se, 0.3 mg; vitamin A, 6000 IU; vitamin D_3,_ 1000 IU; vitamin E, 100 IU; vitamin K, 4 mg; vitamin B_12,_ 15 µg; riboflavin, 2 mg; nicotinic acid, 12 mg; pantothenic acid, 10 mg; choline chloride, 250 mg; vitamin B_1,_ 2 mg; vitamin B_6,_ 3 mg.

6 Premix provided per kg of complete diet: Cu, 15 mg; Fe, 24 mg; Mn, 31 mg; Zn, 80 mg, I, 0.3 mg; Se, 0.2 mg; vitamin A, 2000 IU; vitamin D_3,_ 500 IU; vitamin E, 40 IU; vitamin K, 4 mg; vitamin B_12,_ 15 µg; riboflavin, 2 mg; nicotinic acid, 12 mg; pantothenic acid, 10 mg; vitamin B_1,_ 2 mg; vitamin B_6,_ 3 mg.

7 Formaxol is a blend of encapsulated formic and citric acids and essential oils (Soda Feed Ingredients, Monaco).

8 Mycosorb® is an organic mycotoxin adsorbent (Alltech, Dunboyne, Co. Meath, Ireland).

9 Calculated values.

10 The starter diet was formulated as meal.

### Blood Sampling and Analysis

Blood samples were collected on days 0, 30, 60 and 100 for measurement of immune parameters and on day 110 for the detection of the *cry1Ab* gene and protein. Blood samples were collected from the anterior vena cava of pigs of up to 30 kg body weight, from the external jugular vein for heavier pigs and during exsanguination at slaughter on day 110. Whole blood samples were collected in K_2_EDTA blood collection tubes (Vacuette, Greiner Bio One Ltd, Gloucestershire, UK) and stored at room temperature prior to haematological analysis which was performed within 6 h of collection. Additional whole blood samples were collected in heparinised blood collection tubes (BD Vacutainer Systems, Franklin Lakes, NJ) and stored at room temperature for peripheral blood mononuclear cell (PBMC) isolation. Blood samples were also collected in serum collection tubes (BD Vacutainer Systems, Franklin Lakes, NJ) and centrifuged at 2500×g for 20 min within 3 h to obtain serum. Serum was stored at –20°C for subsequent analysis of Cry1Ab-specific antibodies. Blood samples taken at slaughter (day 110) were collected in K_2_EDTA blood collection tubes and immediately placed on ice for transport to the laboratory. Within 3 h of collection, blood samples were centrifuged at 2500×g for 20 min, after which the buffy coat of white blood cells was removed and stored at –20°C for subsequent tracking of the *cry1Ab* gene. Serum samples were also taken at slaughter as described above for Cry1Ab-specific antibody analysis.

### Digesta and Organ Sampling

On day 110, all pigs were sacrificed by electrical stunning followed by exsanguination. The last meal was administered 3 h prior to slaughter. During sampling, the following precautions were taken to prevent any cross contamination between the Bt and isogenic maize-fed pigs; all isogenic maize-fed pigs were slaughtered first followed by the Bt maize-fed pigs; all surgical instruments were cleaned with 70% ethanol between each animal and all assistants wore single-use gloves that were replaced after each sample was taken; a sample of semi-tendinosus muscle was taken and the liver and kidneys were removed first, to prevent contamination with digesta contents, followed by the entire GIT. Once removed, the liver and kidneys were trimmed of any superficial fat or blood clots. The outermost layer of each tissue was removed to enable sampling of the interior in order to prevent contamination by feed residue. Samples were taken from the semi-tendinosus muscle, liver (centre of quadrate lobe) and kidney (cortex and medulla), snap frozen in liquid N and stored at –20°C for subsequent analysis of the Bt toxin and *cry1Ab* gene. Digesta was then sampled from the stomach (cardiac region), ileum (15 cm distal to the ileo-cecal junction), cecum (tip of the blind end) and colon (60 cm from the rectum) and stored at –20°C for subsequent analysis of the truncated *cry1Ab* gene and Bt toxin.

### Isolation and Stimulation of PBMC and Cytokine Measurement

Isolation and stimulation of PBMC from whole blood was conducted as described by Walsh *et al*. [6], [15]. Following PBMC stimulation, the cell culture supernatant was collected and stored at −80°C. Concentrations of IL-4, IL-6, IL-8, and TNFα were subsequently determined in the cell supernatants using multiplex porcine-specific cytokine ELISA kits (Meso Scale Discovery, Gaithersburg, Maryland) in accordance with the manufacturer’s instructions.

### Immune Cell Phenotyping and Cry1Ab-specific Antibody Response

Following stimulation, PBMC were resuspended at ∼2×10^6^ cells/mL in phosphate buffered saline (PBS) containing 2% fetal bovine serum (PBS-FBS). Primary and secondary antibodies were added at concentrations determined by titration and incubated in the dark at room temperature for 15 min. Cells were washed and re-suspended in PBS-FBS and acquired using a BD FACSCanto II™ flow cytometer. Antibodies used included anti-porcine CD3 PE/Cy5 (Abcam, Cambridge, UK), anti-porcine CD4 fluorescein isothiocyanate (FITC), anti-porcine CD8 phycoerythrin (PE), and anti-mouse CD32 (all antibodies were obtained from BD Biosciences, Devon, UK unless otherwise stated). Antibodies were used according to manufacturer’s recommendations. The percentages of CD4^+^, CD8^+^ and CD4^+^CD8^+^ T lymphocytes were calculated on the CD3^+^ gate. At least 50,000 events were acquired and analyzed. Data were analyzed using FACSDIVA software (BD Biosciences).

The detection of Cry1Ab-specific IgA and IgG in pig serum was conducted as previously described by Walsh *et al*. [6] on samples taken on days 0, 30, 60, 100 and 110.

### Haematological Analysis

Whole blood samples were analyzed using a Beckman Coulter Ac T Diff haematology analyzer (Beckman Coulter Ltd., High Wycombe, UK). The following parameters were determined; counts of white blood cells (WBC), lymphocytes (LY), monocytes (MO), granulocytes (GR), and red blood cells (RBC), haemoglobin concentration (Hgb), haematocrit (Hct), mean corpuscular volume (MCV), mean corpuscular haemoglobin (MCH), mean corpuscular haemoglobin concentration (MCHC), red cell distribution width (RDW), platelet count (Plt) and mean platelet volume (MPV). Samples were analyzed according to the manufacturer’s instructions and general haematology recommendations [16].

### Tracking of the Truncated Bt toxin and *cry1Ab* gene in Feed and Porcine Digesta, Organs and Blood

#### Bt toxin quantification

Organ samples (liver, muscle and kidney) were homogenised in 0.8% saline (0.5 g/mL) and serum was diluted in 0.8% saline (0.5 mL/mL). Ten µL of 10 mM phenylmethylsulfonyl fluoride (PMSF) was added per mL of solution and samples were centrifuged for 20 min at 9390×g. Digesta samples were centrifuged for 15 min at 540×g and 10 µL of 10 mM PMSF was added per mL of supernatant and samples were centrifuged for 20 min at 9390×g. The concentration of the Bt toxin in both the organ and digesta samples was determined as previously outlined by Walsh *et al*. [6].

#### Detection of the *cry1Ab* gene

DNA extraction from digesta, animal tissue and white blood cells was conducted as previously outlined [6]. A preliminary cross-dilution assay was performed to determine the detection limit of the *cry1Ab-*specific PCR and the possible inhibitory effect of porcine DNA. Five primer pairs targeting two endogenous maize genes [*rubisco* and shrunken 2 (*sh2*)], two *cry1Ab* gene fragments (*cry1Ab*-1 and *cry1Ab*-2) and a porcine growth hormone gene (*sw*), respectively were obtained from Invitrogen (Paisley, UK). The primer sequences and PCR conditions used for the detection of *sh2*, *cry1Ab*-1 and *sw* have previously been described by Walsh *et al*. [6]. The primers and conditions used for the detection of *rubisco* and *cry1Ab-*2 are outlined in Table 2. Two microlitres of extracted DNA was used in all PCR reactions, which were performed in a final volume of 50 µL. Each PCR reaction contained 25 µL of either REDTaq ReadyMix PCR reaction mix containing MgCl_2_ (Sigma-Aldrich) (for white blood cells) or DreamTaq Green PCR master mix (Fermentas, Ontario, Canada) (for tissue samples and digesta), as well as 0.6 or 0.1 µM of the *cry1Ab-2* or *rubisco* primers, respectively. PCR reactions were performed in a GeneAmp 2400 or 2700 thermal cycler (Applied Biosystems, Foster City, CA). Each set of PCR reactions included a positive control for the *cry1Ab* gene (DNA from Bt maize), a negative control for the *cry1Ab* and endogenous maize genes (DNA from isogenic maize), contamination controls without template DNA, and a positive control for the endogenous porcine gene (DNA from normal pig meat). PCR products were analyzed on 10% polyacrylamide gels run at 200 V for 50 min and visualized by SYBR Green-staining.

**Table 2:** Primers and PCR conditions used for the detection of target genes in porcine organ, white blood cell and digesta samples.

Primer name	Sequence (5′-3′)	Specificity	Target gene	Amplicon size(bp)	PCR conditions^1^	Ref.
*rubisco* – F	AGC TAA TCG TGT GGC TTT AGA AGC C	Plant (endogenous)	Ribulose bisphosphate carboxylase	173	94°C×3 min94°C×30 s62°C×30 s72°C×30 s72°C×7 min	Guertler *et al.,* 2009
*rubisco* – R	TGG TAT CCA TCG CTT TGA AAC CA	Plant (endogenous)	Ribulose bisphosphate carboxylase		94°C×3 min94°C×30 s62°C×30 s72°C×30 s72°C×7 min	
*cry1Ab-2*– F	ACT ATC CTT CGC AAG ACC CTT CCT C	Plant (transgenic)	*cry1Ab*	149	95°C×3 min95°C×25 s62°C×30 s72°C×45 s72°C×7 min	Nemeth *et al*., 2004
*cry1Ab-2*– R	GCA TTC AGA GAA ACG TGG CAG TAA C	Plant (transgenic)	*cry1Ab*		95°C×3 min95°C×25 s62°C×30 s72°C×45 s72°C×7 min	

1 PCR conditions for *rubisco* F & R included 1 cycle at 94°C for 3 min, 35 cycles of 94°C for 30 s, down to 62°C for 30 s and back up to 72°C for 30 s and 1 cycle of 72°C for 7 min; *cry1Ab*-2**F & R; 1 cycle at 95°C for 3 min, 30 cycles of 95°C for 25 s, down to 62°C for 30 s and back up to 72°C for 45 s and 1 cycle of 72°C for 7 min.

### Statistical Analysis

For all response criteria, the pig was the experimental unit. Immune cell phenotype and haematology data were analyzed as repeated measures using the MIXED procedure of SAS (SAS Inst. Inc., Cary, NC) with Tukey-Kramer adjustment for multiple comparisons using day 0 values as a covariate in the model. Cytokine data found not to be normally distributed following log transformation were analyzed using the non-parametric Kruskal-Wallis test within the NPAR1WAY procedure in SAS. Cytokine data are presented as treatment median values with the 25–75^th^ percentiles. The level of significance for all tests was *P*<0.05 and trends were reported for 0.05<*P*<0.10.

## Results

### Effect of Feeding Bt and Isogenic Maize on Immune Response in Growing Pigs

#### Cytokine production

In the absence of exogenous stimuli, the spontaneous production of IL-6, IL-4 and IL-8 by resting PBMC from pigs on all four dietary treatments was comparable on days 0, 30 and 100 (Table 3). On day 0, (prior to commencement of dietary treatments), PBMC from pigs subsequently fed Bt maize for 110 days produced lower concentrations of TNFα than those from pigs on all other treatments (*P*<0.01). However, TNFα production by resting PBMC was not different between treatments on day 30 or 100 of the study. The concentration of IL-6, IL-4, IL-8 and TNFα produced by mitogen-stimulated PBMC on day 0, 30 and 100 did not differ between treatments (Table 4).

**Table 3:** Cytokine production by resting PBMC from pigs fed diets containing Bt and isogenic maize (pg/mL)^1^.

Cytokine	Isogenic^2^	Bt^3^	Isogenic/Bt^4^	Bt/isogenic^5^	*P*-value
Day 0					
IL-6	4.5	1.2	6.5	4.0	0.16
	(0.1–8.4)	(0.2–15.1)	(1.4–61.1)	(2.5–8.8)	
IL-4	11.8	3.5	8.9	9.4	0.42
	(1.8–57.2)	(0.6–21.3)	(0.2–14.0)	(4.6–22.7)	
IL-8	44.7	69.6	121.0	109.4	0.44
	(8.59–435.42)	(2.1–129.8)	(25.3–5277.9)	(5.5–1497.9)	
TNFα	2.6^x^	0.6^y^	2.4^x^	2.9^x^	0.01
	(0.40–15.84)	(0.2–1.3)	(0.5–20.0)	(1.3–4.9)	
Day 30					
IL-6	2.7	2.2	6.8	4.4	0.14
	(0.3–15.8)	(0.1–6.9)	(2.9–82.0)	(0.3–8.8)	
IL-4	7.2	7.9	9.7	6.1	0.45
	(1.8–24.6)	(5.1–18.2)	(4.5–34.9)	(2.4–14.2)	
IL-8	150.9	381.0	703.7	448.6	0.35
	(95.8–2428.5)	(72.3–2349.1)	(175.1–5415.5)	(33.9–1177.5)	
TNFα	4.1	6.9	7.8	6.7	0.65
	(0.7–22.1)	(0.4–17.6)	(3.6–79.0)	(1.7–18.6)	
Day 100					
IL-6	0.4	1.2	4.5	1.5	0.28
	(0.04–1.7)	0.03–9.7	0.5–8.4	(0.5–2.0)	
IL-4	3.5	6.7	3.0	2.6	0.12
	(0.31–7.18)	(2.8–35.9)	(1.3–5.8)	(0.5–14.3)	
IL-8	553.8	150.1	787.4	354.9	0.14
	(142.1–2109.8)	(11.6–6428.9)	(270.3–2807.1)	(57.1–2397.7)	
TNFα	1.3	2.1	1.4	1.0	0.94
	(0.4–5.9)	(0.1–13.0)	(0.3–10.4)	(0.2–4.8)	

1 Values are given as the median with 25^th^ to 75^th^ percentiles in parentheses.

2 Isogenic: isogenic maize diet for 110 days.

3 Bt: Bt maize diet for 110 days.

4 Isogenic/Bt: isogenic maize diet for 30 days followed by Bt maize diet for 80 days.

5 Bt/isogenic: Bt maize diet for 30 days followed by isogenic maize diet for 80 days.

xy Within a row means without a common superscript differ by *P*<0.05 by means separation using Tukey-Kramer adjustment for multiple comparisons.

**Table 4:** Cytokine production by mitogen-stimulated PBMC from pigs fed diets containing Bt and isogenic maize (pg/mL)^1^.

Cytokine	Isogenic^2^	Bt^3^	Isogenic/Bt^4^	Bt/isogenic^5^	*P*-value
Day 0					
IL-6	18.8	34.9	23.2	112.0	0.84
	(10.3–550.3)	(1.0–416.1)	(3.19–268.6)	(4.1–454.9)	
IL-4	24.0	38.8	34.3	65.1	0.96
	(6.8–148.9)	(13.9–82.5)	(1.8–205.9)	(4.33–125.8)	
IL-8	5438.2	10696.7	9516.9	11739.5	0.89
	(1435.5–22783.7)	(565.9–21144.7)	(469.4–21224.0)	(317.4–27328.6)	
TNFα	43.7	66.9	58.7	125.2	0.85
	(24.2–543.1)	(6.9–304.9)	(7.1–367.1)	(1.3–480.5)	
Day 30					
IL-6	23.3	30.6	19.6	65.9	0.50
	(8.7–91.6)	(11.1–49.7)	(11.3–66.9)	(4.7–134.6)	
IL-4	23.4	33.1	18.9	30.9	0.87
	(5.8–149.1)	(12.1–80.8)	(8.9–108.8)	(10.5–141.1)	
IL-8	4080.5	4009.9	2576.3	5985.2	0.46
	(789.3–13561.9)	(748.9–12187.9)	(373.3–9668.0)	(923.1–12288.2)	
TNFα	30.5	38.3	26.7	55.9	0.72
	(13.8–120.4)	(15.7–80.2)	(12.3–115.6)	(14.9–169.1)	
Day 100					
IL-6	2.9	2.2	6.5	4.8	0.44
	(0.3–7.7)	(0.4–8.7)	(0.3–19.1)	(1.4–15.1)	
IL-4	3.2	9.9	7.6	2.7	0.17
	(1.3–8.7)	(5.1–33.8)	(0.03–38.8)	(1.0–54.2)	
IL-8	2266.3	1838.2	2447.0	3440.3	0.59
	(812.7–6732.5)	(345.6–9734.9)	(1383.6–10625.4)	(398.0–23765.5)	
TNFα	8.6	10.7	17.1	9.0	0.52
	(4.4–18.2)	(9.2–21.0)	(3.9–24.9)	(1.9–34.3)	

1 Values are given as the median with 25^th^ to 75^th^ percentiles in parentheses.

2 Isogenic: isogenic maize diet for 110 days.

3 Bt: Bt maize diet for 110 days.

4 Isogenic/Bt: isogenic maize diet for 30 days followed by Bt maize diet for 80 days.

5 Bt/isogenic: Bt maize diet for 30 days followed by isogenic maize diet for 80 days.

#### Immune cell populations

Treatment×time interactions for all immune cell populations examined were non-significant (Table 5). There was no effect of feeding Bt maize to pigs on populations of CD3^+^, CD3^+^CD4^+^ or CD3^+^CD8^+^ T cells isolated from PBMC at any time during the study. The proportion of CD3^+^ T cells increased in pigs on day 60 of the study; however, by day 100, it was reduced to below day 30 values (*P*<0.05). The number of both CD3^+^CD4^+^ and CD3^+^CD8^+^ T cells decreased on day 60 of the study but increased again by day 100 (*P*<0.001). On day 30, pigs fed isogenic maize for 30 days followed by Bt maize for 80 days tended to have a greater proportion of CD4^+^CD8^+^ T cells than pigs fed isogenic maize (*P* = 0.08) but cell proportions in these pigs did not differ from the other two treatments. Similar to findings for both CD3^+^CD4^+^ and CD3^+^CD8^+^ T cells, the proportion of CD4^+^CD8^+^ T cells decreased over time until day 60 and increased thereafter (*P*<0.001).

**Table 5:** Effects of feeding Bt and isogenic maize on immune cell phenotypes of peripheral blood mononuclear cells.

Day	Treatment					Mean	*P* – value		
	Isogenic^1^	Bt^2^	Isogenic/Bt^3^	Bt/isogenic^4^			Treatment	Time	Treatment×Time
	CD3^+^ T cells								
30	55.5±6.63	53.5±5.95	52.1±6.76	49.8±6.51	52.7±3.11		0.94		
60	68.4±6.92	60.4±6.39	62.9±6.71	56.9±7.08	62.2±3.36		0.76		
100	53.5±6.63	43.1±6.31	50.9±7.30	54.9±6.51	50.6±3.35		0.51		
Mean	59.0±4.75	52.3±4.04	55.3±4.56	53.9±4.63			0.82	0.02	0.84
	CD3^+^CD4^+^ T cells								
30	16.1±2.65	18.3±2.36	19.7±2.64	20.5±2.48	18.7±1.20		0.68		
60	5.2±2.61	7.6±2.44	8.6±2.63	5.7±2.59	6.7±1.27		0.77		
100	10.8±2.65	11.4±2.45	11.0±2.82	10.3±2.48	10.9±1.27		0.99		
Mean	10.7±2.14	12.4±1.89	13.1±2.14	12.2±2.02			0.90	0.001	0.82
	CD3^+^CD8^+^ T cells								
30	26.5±2.37	26.7±2.22	27.6±2.46	31.3±2.45	28.0±1.15		0.45		
60	15.1±2.36	12.8±2.34	11.2±2.46	12.2±2.56	12.9±1.24		0.70		
100	24.0±2.37	20.2±2.34	25.3±2.66	22.1±2.45	22.9±1.24		0.52		
Mean	21.9±1.62	19.9±1.55	21.4±1.68	21.9±1.76			0.78	0.001	0.40
	CD4^+^CD8^+^ T cells								
30	7.3^b^±1.21	8.8^ab^±1.14	11.8^a^±1.26	9.6^ab^±1.19	9.4±0.59		0.08		
60	4.0±1.20	5.2±1.21	3.5±1.26	2.6±1.26	3.8±0.63		0.48		
100	3.9±1.21	4.2±1.20	4.7±1.36	4.0±1.19	4.2±0.63		0.97		
Mean	5.1±0.81	6.1±0.79	6.7±0.85	5.4±0.81			0.51	0.001	0.29

The CD4^+^, CD8^+^, CD4^+^CD8^+^ immune cell populations are given as proportions of CD3^+^ T cells (%). All values are shown ±SE.

1 Isogenic: isogenic maize diet for 110 days.

2 Bt: Bt maize diet for 110 days.

3 Isogenic/Bt: isogenic maize diet for 30 days followed by Bt maize diet for 80 days.

4 Bt/isogenic: Bt maize diet for 30 days followed by isogenic maize diet for 80 days.

ab Within a row means without a common superscript differ by *P*<0.10 by means separation using Tukey-Kramer adjustment for multiple comparisons.

#### Cry1Ab-specific immunoglobulin production

Cry1Ab-specific IgA and IgG were not detected in the serum of pigs fed either Bt or isogenic maize at any time point during the study (day 0, 30, 60, 100) or at slaughter on day 110 or in non-maize-fed pigs (control) even at the lowest dilution (data not shown).

#### Haematological parameters

Overall, the total count of leukocytes was not altered by dietary treatment. However, on day 100, pigs fed Bt/isogenic maize diets tended to have higher leukocyte counts than pigs fed isogenic or Bt maize diets for 110 days (Table 6; *P* = 0.06). Furthermore, leukocyte counts increased as the pigs aged (*P*<0.001). Overall, there was a tendency for higher lymphocyte counts in pigs fed Bt/isogenic maize diets compared with pigs fed isogenic or Bt maize diets for the entire 110 day period (*P* = 0.09). This was brought about by similar pattern of differences, this time significant, on day 100 (*P*<0.05). Monocyte counts tended to be higher on day 100 in pigs fed Bt/isogenic maize diets or isogenic/Bt maize diets compared with pigs fed Bt maize for 110 days (*P* = 0.09). Overall, there were no dietary treatment effects on monocyte counts; however, counts increased over time (*P*<0.01). Granulocyte counts were not altered by dietary treatment; however, an initial decrease in granulocyte count was followed by an overall increase by day 100 of the study (*P*<0.01).

**Table 6:** Effects of feeding Bt and isogenic maize on immune cell counts (×1000/µL) ±SE in growing pigs.

Day	Treatment							Mean	*P* - value		
	Isogenic^1^		Bt^2^		Isogenic/Bt^3^		Bt/isogenic^4^		Treatment	Time	Treatment×Time
	Leukocytes^5^										
30	18.0±2.96		22.0±2.44		25.1±2.54		26.4±2.71	22.9±1.20	0.28		
60	19.5±2.80		23.6±2.72		25.8±2.80		28.4±2.71	24.3±1.34	0.25		
100	22.7^b^±2.70		28.6^b^±2.56		29.5^ab^±2.80		35.2^a^±2.71	29.0±1.26	0.06		
Mean	20.0±2.26		24.7±1.86		26.8±2.01		30.0±2.12		0.13	0.001	0.95
	Lymphocytes^6^										
30	12.9±1.86	14.7±1.73			15.4±1.74		17.1±1.80	15.0±0.85	0.49		
60	13.5±1.80	16.1±1.93			18.5±1.87		18.9±1.180	16.7±0.94	0.19		
100	13.8^y^±1.71	15.4^y^±1.81			17.9^xy^±1.87		21.4^x^±1.80	17.1±0.87	0.04		
Mean	13.4^c^±1.30	15.4^bc^±1.28			17.2^ab^±1.25		19.1^a^±1.30		0.09	0.15	0.87
	Monocytes^7^										
30	1.7±0.23		1.4±0.22		1.7±0.22		1.6±0.22	1.61±0.10	0.78		
60	1.6±0.23		1.7±0.25		1.9±0.23		1.9±0.22	1.79±0.12	0.76		
100	2.2^ab^±0.21		1.7^b^±0.23		2.3^a^±0.23		2.5^a^±0.22	2.17±0.11	0.09		
Mean	1.84±0.14		1.60±0.14		1.98±0.13		2.00±0.13		0.21	0.002	0.77
	Granulocytes										
30	2.6±2.5	6.8±1.84		8.5±1.97		8.1±2.17		6.5±0.90	0.45		
60	3.5±2.45	6.7±2.02		5.6±2.15		8.1±2.17		6.0±0.10	0.64		
100	5.9±2.39	12.4±1.91		8.8±2.15		11.9±2.17		9.7±0.94	0.24		
Mean	3.7±2.14	8.7±1.45		7.6±1.64		9.3±1.81			0.45	0.002	0.68

1 Isogenic: isogenic maize diet for 110 days.

2 Bt: Bt maize diet for 110 days.

3 Isogenic/Bt: isogenic maize diet for 30 days followed by Bt maize diet for 80 days.

4 Bt/isogenic: Bt maize diet for 30 days followed by isogenic maize diet for 80 days.

5 Normal reference range in pigs = 11,000–22,000/µL [20].

6 Normal reference range in pigs = 4,300–13,600/µL [20].

7 Normal reference range in pigs = 200–2,200/µL [20].

xy Within a row means without a common superscript differ by *P*<0.05 by means separation using Tukey-Kramer adjustment for multiple comparisons.

abc Within a row means without a common superscript differ by *P*<0.10 by means separation using Tukey-Kramer adjustment for multiple comparisons.

On day 100, pigs fed Bt/isogenic maize diets had higher erythrocyte counts compared with pigs fed isogenic/Bt maize diets or Bt maize for the entire 110 days (Table S1; *P*<0.05). Overall, however, there were no dietary treatment effects on erythrocyte counts. In addition, erythrocyte counts were found to increase up to day 60 of the study and to decrease thereafter (*P*<0.01). Haemogloblin concentration, haematocrit or the erythrocyte parameters MCV, MCH, MCHC and RDW were not altered by dietary treatment. Likewise, platelet counts and MPV were not affected but were lower in older pigs (*P*<0.001). The erythrocyte indices MCH (*P*<0.01), and MCHC (*P*<0.001) increased over time, while MCV was lower in older pigs (*P*<0.001). The erythrocyte index RDW also tended to increase over time (*P* = 0.07). Both haemogloblin concentration and hematocrit initially increased as the pigs aged but decreased thereafter (*P*<0.01).

### Fate of Ingested *cry1Ab* gene and Truncated Bt Toxin

#### Detection of transgenic and endogenous plant genes in white bloods cells, tissue and digesta

Neither transgenic *cry1Ab* plant gene fragments of 211 or 149 bp were detected in liver, kidneys, muscle or in white blood cells of any pigs, regardless of dietary treatment (Table 7). Likewise, fragments of a single copy endogenous plant gene (*sh2)* were not detected in any tissue or white blood cells examined. However, a multiple copy endogenous plant gene (*rubisco*) was detected in 40–60% of liver samples, 30–50% of kidney samples, 20–50% of muscle samples and 0–20% of white blood cells examined, depending on treatment. All tissue and white blood cell samples were positive for the endogenous porcine gene (*sw*). The single copy endogenous plant gene, *sh2* was detected in the gastric digesta of 80–90% of pigs, depending on treatment but was undetectable in the ileal, cecal and colon digesta (Table 8). Likewise, the multiple copy *rubisco* gene was found in 90–100% of gastric digesta samples, depending on treatment. However, unlike the *sh2* gene, *rubisco* was detected in 80–100% of ileal, 30–60% of cecal and 10–40% of colon digesta samples, depending on treatment. Both transgenic plant gene fragments (*cry1Ab-1 and cry1Ab-2*) were found in the gastric digesta of 90% of the pigs fed Bt maize either for the entire 110 days or only for the final 80 days of the study. Faint signal bands for both *cry1Ab-1* and *cry1Ab-2* were detected in gastric digesta of 40% of the pigs fed either isogenic or Bt/isogenic maize diets, respectively at slaughter on day 110 (data not shown). This is thought to have occurred due to sample contamination post-sampling. No Bt toxin fragments were detected in gastric digesta from these pigs. Likewise, *cry1Ab* gene contamination was not found in other digesta samples from these pigs and prior to feeding, no *cry1Ab* gene fragments were detected in the isogenic maize [12]. The *cry1Ab*-1 gene fragment was detected in the ileal digesta of 20 and 10% of the pigs fed Bt maize for the entire 110 day period and the final 80 days of the study, respectively. However, the *cry1Ab*-2 gene fragment was not detected in the ileal digesta of any pigs fed Bt maize at any time. Likewise, no transgenic plant gene fragments were detected in the cecal or colon digesta of pigs fed Bt maize for 80 or 110 days. All digesta samples from pigs fed the isogenic maize diet for 110 days or Bt/isogenic maize diets did not contain either transgenic plant gene fragment.

**Table 7:** Detection of endogenous maize and porcine genes and transgenic *cry1Ab* gene in the organs and blood of pigs fed Bt and isogenic maize^1^.

Fragment amplified	Organ															
	Liver				Kidney				Muscle				White blood cells			
Dietary treatment^2^	1	2	3	4	1	2	3	4	1	2	3	4	1	2	3	4
**Endogenous**																
*Sh2* (maize)	0	0	0	0	0	0	0	0	0	0	0	0	0	0	0	0
*rubisco* (maize)	6	5	4	5	4	3	3	5	3	2	5	4	2	2	0	1
*sw* (porcine)	10	10	10	10	10	10	10	10	10	10	10	10	10	10	10	10
**Transgenic**																
*cry1Ab-1* (211 bp; maize)	0	0	0	0	0	0	0	0	0	0	0	0	0	0	0	0
*cry1Ab-2* (149 bp; maize)	0	0	0	0	0	0	0	0	0	0	0	0	0	0	0	0

1 Number of samples that tested positive for the gene of interest out of 10 samples analyzed. One sample was tested per pig (*n* = 10 pigs per treatment).

2 Dietary treatments; 1) isogenic maize diet for 110 days, 2) Bt maize diet for 110 days, 3) isogenic maize diet for 30 days followed by Bt maize diet for 80 days and 4) Bt maize diet for 30 days followed by isogenic maize diet for 80 days.

**Table 8:** Detection of endogenous maize and porcine genes and transgenic *cry1Ab* gene in stomach, ileum, cecum and colon digesta of pigs fed Bt and isogenic maize diets^1^.

Fragment amplified	Stomach				Ileum				Cecum				Colon			
Dietary treatment^2^	1	2	3	4	1	2	3	4	1	2	3	4	1	2	3	4
**Endogenous**																
*sh2* (maize)	9	9	9	8	0	0	0	0	0	0	0	0	0	0	0	0
*rubisco* (maize)	10	9	9	8	10	10	8	9	4	5	6	3	1	1	1	4
**Transgenic**																
*cry1Ab-1* (211 bp; maize)	0	9	9	0	0	2	1	0	0	0	0	0	0	0	0	0
*cry1Ab-2* (149 bp; maize)	0	9	9	0	0	0	0	0	0	0	0	0	0	0	0	0

1 Number of samples that tested positive for the gene of interest out of 10 samples analyzed. One sample was tested per pig (*n* = 10 pigs per treatment).

2 Dietary treatments; 1) isogenic maize diet for 110 days, 2) Bt maize diet for 110 days, 3) isogenic maize diet for 30 days followed by Bt maize diet for 80 days and 4) Bt maize diet for 30 days followed by isogenic maize diet for 80 days.

#### Detection of the transgenic Bt toxin in serum, tissue and digesta

The Bt toxin was not detected in the kidneys, liver, muscle or in the sera of any of the pigs fed any of the four dietary treatments at any time point during the study (data not shown). The Bt toxin was not detected in the stomach, cecal or colon digesta of pigs fed the isogenic maize diet or pigs fed the Bt/isogenic maize diet. It was only detected in the digesta of pigs fed Bt maize for 110 days or the isogenic/Bt maize diet (Table 9). In these pigs, it was detected in 90 and 80% of the gastric samples, 80 and 50% of cecal samples and 100% of colon samples, respectively 3 h after the last meal was administered. The mean concentration of Bt toxin was lower in the digesta of pigs fed the isogenic/Bt maize diet compared with those fed Bt maize for the entire study period, except in the cecum where the opposite was true. In both Bt maize-fed groups, the mean concentration of Bt toxin in the cecal digesta was lower than in the gastric or colon digesta (Table 9). In fact, the Bt toxin was most concentrated in the colon digesta.

**Table 9:** Mean Bt toxin concentrations (ng/mL) in gastrointestinal digesta of pigs fed Bt and isogenic maize^1^.

Treatment	Isogenic^2^	Bt^3^	Isogenic/Bt^4^	Bt/isogenic^5^
Stomach	BLD^6^ (0)	28.26 (90)	12.97 (80)	BLD (0)
Cecum	BLD (0)	7.92 (80)	11.45 (50)	BLD (0)
Colon	BLD (0)	32.74 (100)	18.05 (100)	BLD (0)

1 Values in parentheses correspond to the percentage of pigs within each treatment that tested positive for the Bt toxin.

2 Isogenic: isogenic maize diet for 110 days.

3 Bt: Bt maize diet for 110 days.

4 Isogenic/Bt: isogenic maize diet for 30 days followed by Bt maize diet for 80 days.

5 Bt/isogenic: Bt maize diet for 30 days followed by isogenic maize diet for 80 days.

6 BLD – below the limit of detection (stomach; 0.82 ng/mL, cecum; 2.16 ng/mL, colon; 1.48 ng/mL).

## Discussion

To our knowledge, this study is the first to evaluate the effects of long-term feeding (80 or 110 days) of Bt maize on peripheral immune response of pigs. It is also the first to investigate if age at feeding impacts the response in pigs. By using a cross-over study, we were able to evaluate any residual effects on peripheral immune response that may emerge in older pigs having received Bt maize for a relatively short time period in early life (post-weaning) as well as the effects of feeding Bt maize for a longer period later in life. Changes in peripheral immune response were evaluated through measurement of cytokine production from PBMC, investigation of Cry1Ab-specific antibody production in serum, immunophenotyping and haematological analysis. In pigs fed Bt maize for the entire 110-day study period and in pigs that were older when first fed Bt maize, there was no change in the production of IL-6, IL-4, IL-8 and TNFα from mitogen-stimulated or resting PBMC between treatments at any time point. TNFα production from resting PBMC isolated from the Bt maize group was less on day 0 than all other treatments. This difference however, cannot be attributed to Bt maize, as these samples were taken prior to feeding of treatment diets. A study examining the effects of glufosinate-ammonium tolerant triticale on the immune system of mice found increased IL-2 and decreased IL-6 in serum but no significant change in IL-4, IL-10, IL-12 or IFNγ concentrations in the fifth generation [17]. Finamore *et al*. [5] reported an age-specific serum cytokine response to feeding MON810 maize in mice where IL-6, IL-13, IL-12p70 and MIP-1ß were elevated in weaning mice fed MON810 maize for 30 days; however, MIP-1ß was the only cytokine elevated in weaning or old mice after 90 days of feeding. Our group previously found that feeding Bt maize to weanling pigs for a shorter time period i.e. 31 days had no effect on the production of IL-10, IL-6, IL-4 or TNFα from mitogen-stimulated or resting PBMC; however a reduction in both IL-12 and IFNγ was observed [6].

In our study, there was no effect of feeding Bt maize for 80 or 110 days on CD3^+^, CD4^+^ and CD8^+^ T cells. On day 30, there tended to be a difference in CD4^+^CD8^+^ T cells between the isogenic and isogenic/Bt groups. However, at this stage both groups were receiving the same diet i.e. non-GM feed; therefore, this difference is not related to Bt maize consumption. In a previous study, feeding weaning mice MON810 maize for 90 days resulted in increased B cells in blood; however, when older mice were fed the same maize for 90 days, B cell and CD8^+^ T cells populations were decreased, while CD4^+^ T cells were increased [5]. Krzyzowska *et al*. [17] reported a decrease in B cells in blood of the fifth generation of mice fed GM triticale. While changes in B cell populations in response to feeding Bt maize were not evaluated in our study, we did not find the changes in CD4^+^ and CD8^+^ T cells observed by others in mice [5].

Our group has previously found no Cry1Ab-specific antibody response in weanling pigs following 31 days of feeding Bt maize [6], which is in agreement with the findings from this study where 80 or 110 days of feeding Bt maize also did not elicit an antigen-specific antibody response. An antigen-specific IgG1 response has been reported in mice fed diets containing rice expressing the Cry1Ab toxin and spiked with the purified Bt toxin for 28 days and a weak specific IgG2a response was evident after feeding rice expressing Bt toxin for 90 days [18]. However, similar to our findings, Adel-Patient *et al*. [19] found no specific anti-Cry1Ab antibody response in serum from mice fed MON810 maize following intragastric or intraperitoneal sensitization.

On day 100, pigs fed the Bt/isogenic maize diet tended to have a higher leukocyte count than those fed either Bt or isogenic maize for 110 days. This increase was primarily a reflection of the increase in lymphocyte count in these pigs. Both leukocyte and lymphocyte counts for all pigs fed Bt maize at some point during the study were above the normal reference range for pigs [20]. The immunophenotyping data indicated that T cell populations were not influenced by feeding Bt maize; however, B cells were not evaluated. While lymphocyte counts were elevated significantly in some pigs fed Bt maize, there was no indication of a Th 2-mediated allergic inflammatory response to the Cry1Ab toxin in the form of antigen-specific Ig production. The spleen weight of these pigs, reported previously by Buzoianu et al. [21], did not differ between treatments and no histopathological indicators of organ damage were evident in the spleen or other organs. Likewise, the cecal bacterial community structure was similar across treatments [22] and as a result alterations in immune response as a consequence of changes in gut microbiota were not anticipated [23]. A study using rats as an animal model for the safety evaluation of Bt rice found that leukocyte count and MCH were decreased in male rats; however, all haematological parameters analyzed were within the reference range for rats of the age and breed used [24]. Krzyzowska *et al*. [17] also found that leukocyte counts were increased when mice were fed GM triticale, but again, these values were within the normal reference range for mice. Erythrocyte counts in pigs fed Bt maize for 80 days or longer were lower than in pigs fed the Bt/isogenic maize diet. We have previously reported a decrease in erythrocyte counts in sows fed Bt maize (Text S1). However, in that study haemogloblin concentration and hematocrit were also decreased and the changes observed were not attributed to Bt maize consumption.

In an earlier study with weanling pigs (∼65 days old), we detected a small (211 bp) fragment of the *cry1Ab* gene in the gastric digesta of all pigs fed Bt maize; however, detection in the ileal and cecal digesta was limited to two and one pigs, respectively while the gene fragment was undetectable in the colon [6]. However, in the present study, in older pigs (∼150 days old) *cry1Ab* gene fragments (149 and 211 bp) were detectable in the gastric digesta and the 211 bp gene fragment only was detected at low frequency in ileal digesta. The *cry1Ab* gene, regardless of amplicon size, was not detected in the cecum or colon. Nucleic acids are known to endure extensive enzymatic degradation in the GIT [25]. Potentially, the transgenic DNA was degraded by microbial DNAse enzymes which are most likely present at higher concentrations in the cecum and colon as a result of larger microbial populations. These findings agree with results from a wild boar study where *cry1Ab* fragments of up to 420 bp were detected in gastric contents but no fragments greater than 211 bp were found further down the GIT [26]. In the same study, a small (173 bp) *rubisco* gene fragment was found throughout the GIT and a small (∼ 100 bp) fragment of *cry1Ab* was found at very low frequency in the jejunal contents [26]. The smallest fragment we chose to detect in our study was 149 bp; therefore, smaller *cry1Ab* fragments may have been present in digesta distal to the stomach but remain undetected. Similar to Wiedemann *et al*. [26], and Chowdhury *et al*. [27], we detected a small (173 bp) *rubisco* fragment in small intestinal, cecal and colon digesta. Chowdhury *et al*. [27], also detected a small (110 bp) *cry1Ab* fragment in 40 kg pigs fed Bt maize for 28 days.

The majority of studies, both in monogastric and ruminant species, have failed to detect transgenic DNA beyond the gastrointestinal barrier [28], [29]. Furthermore, our group were previously unable to detect a 211 bp fragment of the transgenic *cry1Ab* gene in the organs or blood of pigs fed Bt (MON810) maize for 31 days [6]. In agreement with these findings, a longer feeding period of 110 days did not influence the ability of *cry1Ab* to translocate across the intestinal barrier of pigs, as neither the 211 or 149 bp *cry1Ab* fragments were detectable in the blood, liver, muscle, or kidneys in the present study. Mazza *et al*. [11], however, detected a 519 bp *cry1Ab* fragment in the plasma, liver, kidney and spleen of piglets fed Bt (MON810) maize for 35 days, although the gene’s smallest functional unit (1800 bp) was never detected. Likewise, a 278 bp fragment of the *cp4epsps* transgene from Round-up Ready canola was found in the liver and kidneys of pigs, however, the prevalence was extremely low [10]. The transfer of endogenous plant DNA from the GIT into blood and organs appears to occur spontaneously in nature [11], [30], [31]. Our findings are similar to those reported in calves [32] and fallow deer [29] where small fragments of the multiple copy endogenous chloroplast *rubisco* gene (173 or 226 bp) were detected in liver, kidney and spleen. Guertler *et al*. [29] and Mazza *et al*. [11] also detected fragments of the multiple copy endogenous chloroplast *zein* gene in organs of deer and pigs fed Bt maize, respectively. We were previously unable to detect fragments of the single copy endogenous chloroplast *sh*2 gene in the organs or blood of pigs fed Bt maize for 31 days [6] and similar results were found when pigs were fed Bt maize for a longer duration in the present study. These findings suggest that copy number is the rate limiting step in the traceability of transgenic DNA. Also, the low detection frequency of the *cry1Ab* gene in the ileum and the absence of *cry1Ab* gene detection distal to the ileum may account for the lack of detection of the single copy *cry1Ab* gene in animal tissues and blood.

Similar to our findings in weanling pigs [6], the Bt toxin was detected in the stomach, cecum and colon digesta but not in the organs or plasma of pigs fed Bt maize for 110 days. Similarly, in other studies with pigs, Yonemochi *et al*. [7] did not detect the Cry9C protein in blood, liver or muscle samples and Wiedemann *et al*. [26] found the Cry1Ab protein in stomach, colon and rectum samples from wild boar fed Bt176 maize but not in the organs or blood. In the present study, the Cry1Ab protein (Bt toxin) was recovered from 85% of stomach, 65% of cecum and 100% of colon samples from pigs fed Bt maize even though the *cry1Ab* gene (either 211 or 149 bp fragments) was only detected in the stomach and at low frequency in the ileum. Einspanier *et al*. [33] reported that the use of PCR primers for shorter amplicons increased the chance of detecting plant DNA in ruminal contents. Therefore, similar to our previous study [6], the discrepancies observed in detection frequency between the *cry1Ab* gene and Bt toxin fragments along the GIT may be due to the failure to amplify gene fragments of less than 149 bp in length. In addition, in our study, the concentration of Bt toxin found in the stomach and colon digesta was twice as high as that found in the cecum. This is likely due to the fact that the colon digesta is more concentrated as a result of water absorption. Furthermore, the concentration of Bt toxin detected in the digesta from any site within the GIT of finisher pigs in this study is higher than that previously found by our group in weanling pigs (2.41–2.74 ng/mL) [6]. This is most likely due to the higher inclusion of Bt maize in finisher diets compared to those used for weanling pigs. The concentration of Bt toxin in the digesta of pigs fed Bt maize during the entire 110-day study period was higher at all gastrointestinal sites, with the exception of the cecum, than in pigs fed the isogenic/Bt maize diet. The longer duration of feeding may account for this difference. Also, the Bt toxin was no longer present in the digesta of pigs fed the Bt/isogenic diet. This is not surprising, as these pigs had not been fed Bt maize for 80 days. This demonstrates that once Bt maize feeding ceases, the Bt toxin is no longer detectable in the GIT.

In conclusion, long-term feeding of Bt maize to pigs did not elicit an allergic or inflammatory-type peripheral immune response. This was evidenced by the lack of antigen-specific antibody production and the absence of alterations in T cell populations and inflammatory cytokine production. Peripheral immune response to Bt maize did not appear to be age-related, as there were no differences in cytokines, antigen-specific Ig production or T cell populations between pigs fed Bt maize from 40 or 70 days of age. However, a residual effect on lymphocyte count was apparent in older pigs fed Bt maize in early life, an effect which was not present following long-term Bt or isogenic maize consumption. Intestinal degradation of transgenic DNA was enhanced in older pigs, as the *cry1Ab* gene was detected in gastric digesta and at low frequency in the ileum but not in the distal GIT, unlike Bt toxin fragments which were found in the colon. Furthermore, long-term feeding of Bt maize did not result in the translocation of transgenic DNA or Bt toxin from the GIT to organs or blood. Findings from this study can offer assurance to regulators and consumers as to the safety of long-term consumption of Bt maize.

## Supporting Information

Text S1 **Supplemental Information.**
(DOC)

Table S1 **Effects of feeding of Bt and isogenic maize on haematological parameters in growing pigs.**
(DOC)

## References

[1] James C (2011). Global status of commericalized biotech/GM crops:2011..

[2] EFSA (2008). Safety and nutritional assessment of GM plants and derived food and feed: The role of animal feeding trials.. Food Chem Toxicol.

[3] Snell C, Bernheim A, Berge J-B, Muntz M, Pascal G (2012). Assessment of the health impact of GM plant diets in long-term and multigenerational animal feeding trials: A literature review.. Food Chem Toxicol.

[4] Prescott VE, Campbell PM, Moore A, Mattes J, Rothenberg ME (2005). Transgenic expression of bean α-amylase inhibitor in peas results in altered structure and immunogenicity.. J Agri Food Chem.

[5] Finamore A, Roselli M, Britti S, Monastra G, Ambra R (2008). Intestinal and peripheral immune response to MON810 maize ingestion in weaning and old mice.. J Agric Food Chem.

[6] Walsh MC, Buzoianu SG, Gardiner GE, Rea MC, Gelencser E (2011). Fate of transgenic DNA from orally administered Bt MON810 maize and effects on immune response and growth in pigs.. PLoS ONE.

[7] Yonemochi C, Suga K, Harada C, Hanazumi M (2010). Tevaluation of transgenic event CBH 351 (StarLink) corn in pig.. Anim Sci J.

[8] Alexander TW, Reuter T, Okine E, Sharma R, McAllister TA (2006). Conventional and real-time polymerase chain reaction assessment of the fate of transgenic DNA in sheep fed Roundup Ready rapeseed meal.. Br J Nutr.

[9] Deaville ER, Maddison BC (2005). Detection of transgenic and endogenous plant DNA fragments in the blood, tissues, and digesta of broilers.. J Agric Food Chem.

[10] Sharma R, Damgaard D, Alexander TW, Dugan ME, Aalhus JL (2006). Detection of transgenic and endogenous plant DNA in digesta and tissues of sheep and pigs fed Roundup Ready canola meal.. J Agric Food Chem.

[11] Mazza R, Soave M, Morlacchini M, Piva G, Marocco A (2005). Assessing the transfer of genetically modified DNA from feed to animal tissues.. Transgenic Res.

[12] Walsh MC, Buzoianu SG, Gardiner GE, Rea MC, Ross RP (2012). Effects of short-term feeding of Bt MON810 maize on growth performance, organ morphology and function in pigs.. Br J Nutr.

[13] NRC (1998). Nutrient requirements of swine: National Academy Press.

[14] Lawlor P, Lynch P, Caffrey P, O Doherty J (2003). The effect of choice feeding complete diets on the performance of weaned pigs.. Anim Sci.

[15] Walsh MC, Gardiner GE, Hart OM, Lawlor PG, Daly M (2008). Predominance of a bacteriocin-producing *Lactobacillus salivarius* component of a five-strain probiotic in the porcine ileum and effects on host immune phenotype.. FEMS Microbiol Ecol.

[16] Feldman BF, Zinkl JG, Jain NC, Schalm OW (2000). Schalm’s Veterinary Hematology: Lippincott Williams & Wilkins.

[17] Krzyzowska M, Wincenciak M, Winnicka A, Baranowski A, Jaszczak K (2010). The effect of multigenerational diet containing genetically modified triticale on immune system in mice.. Polish J Vet Sci.

[18] Kroghsbo S, Madsen C, Poulsen M, Schrøder M, Kvist PH (2008). Immunotoxicological studies of genetically modified rice expressing PHA-E lectin or Bt toxin in Wistar rats.. Toxicol.

[19] Adel-Patient K, Guimaraes VD, Paris A, Drumare M-F, Ah-Leung S (2011). Immunological and metabolomic impacts of administration of Cry1Ab protein and MON810 maize in mouse.. PLoS ONE.

[20] Thorn CE (2000). Normal hematology of the pig..

[21] Buzoianu SG, Walsh MC, Rea MC, Ross RP, Gardiner GE (2012). Effect of feeding Bt MON810 maize to ∼40 day old pigs for 110 days on growth and health indicators..

[22] Buzoianu SG, Walsh MC, Rea MC, O’Sullivan O, Crispie F (2012). The effect of feeding Bt MON810 maize to pigs for 110 days on intestinal microbiota..

[23] Festi D, Schiumerini R, Birtolo C, Marzi L, Montrone L (2011). Gut microbiota and its pathophysiology in disease paradigms.. Dig Dis.

[24] Schroder M, Poulsen M, Wilcks A, Kroghsbo S, Miller A (2007). A 90-day safety study of genetically modified rice expressing Cry1Ab protein (*Bacillus thuringiensis* toxin) in Wistar rats.. Food Chem Toxicol.

[25] Phipps RH, Beevers DE (2000). New technology. Issues relating to the use of genetically modified crops.. J Anim Feed Sci.

[26] Wiedemann S, Lutz B, Albrecht C, Kuehn R, Killermann B (2009). Fate of genetically modified maize and conventional rapeseed, and endozoochory in wild boar (*Sus scrofa*).. Mamm Biol.

[27] Chowdhury EH, Kuribara H, Hino A, Sultana P, Mikami O (2003). Detection of corn intrinsic and recombinant DNA fragments and Cry1Ab protein in the gastrointestinal contents of pigs fed genetically modified corn Bt11.. J Anim Sci.

[28] Flachowsky G, Chesson A, Aulrich K (2005). Animal nutrition with feeds from genetically modified plants.. Arch Anim Nutr.

[29] Guertler P, Lutz B, Kuehn R, Meyer H, Einspanier R (2008). Fate of recombinant DNA and Cry1Ab protein after ingestion and dispersal of genetically modified maize in comparison to rapeseed by fallow deer (*Dama dama*).. Eur J Wildlife Res.

[30] Nemeth A, Wurz A, Artim L, Charlton S, Dana G (2004). Sensitive PCR analysis of animal tissue samples for fragments of endogenous and transgenic plant DNA.. J Agric Food Chem.

[31] Reuter T, Aulrich K (2003). Investigations on genetically modified maize (Bt-maize) in pig nutrition: fate of feed-ingesting foreign DNA bodies.. Eur Food Res Technol.

[32] Chowdhury EH, Mikami O, Murata H, Sultana P, Shimada N (2004). Fate of maize intrinsic and recombinant genes in calves fed genetically modified maize Bt11.. J Food Prot.

[33] Einspanier R, Lutz B, Rief S, Berezina O, Zverlov V (2004). Tracing residual recombinant feed molecules during digestion and rumen bacterial diversity in cattle fed transgenic maize.. Eur Food Res Technol.

