# Supplementary material for: Effects of Feeding Bt MON810 Maize to Pigs for 110 Days on Peripheral Immune Response and Digestive Fate of the cry1Ab Gene and Truncated Bt Toxin
Source: PLoS One. 2012 May 4;7(5):e36141. doi: 10.1371/journal.pone.0036141 (PMC3345032; doi:10.1371/journal.pone.0036141)
Supplement: Text S1 — Supplemental Information. (DOC) [file pone.0036141.s001.doc]

**Effects of feeding Bt MON810 maize to sows during first gestation and lactation on maternal and offspring health**

Maria C. Walsh^1^, Stefan G. Buzoianu^1,2^, Gillian E. Gardiner^2^, Mary C. Rea^3^, Orla O’Donovan^2^, R. Paul Ross^3,4^, and Peadar G. Lawlor^1*^

^1^Teagasc, Pig Development Department, Animal and Grassland Research and Innovation Centre, Moorepark, Fermoy, Co. Cork, Republic of Ireland

^2^Department of Chemical and Life Sciences, Waterford Institute of Technology, Waterford, Republic of Ireland

^3^Teagasc, Food Research Centre, Moorepark, Fermoy, Co. Cork, Republic of Ireland

^4^Alimentary Pharmabiotic Centre, University College Cork, Cork, Republic of Ireland

*Corresponding author: P. G. Lawlor, Teagasc, Pig Development Department, Animal and Grassland Research and Innovation Centre, Moorepark, Fermoy, Co. Cork, Republic of Ireland Tel.: +353 25 42217; Fax: +353 25 42340; email: [peadar.lawlor@teagasc.ie](mailto:peadar.lawlor@teagasc.ie).

Running heading; Feeding genetically modified Bt maize to sows

**KEYWORDS;** Corn, cry1Ab, multigeneration, genetically modified, safety

**ABSTRACT**

Twenty-four sows and their offspring were used in a 20-week study to investigate the effects of feeding genetically modified (GM) maize on maternal and offspring health. Sows were fed diets containing GM or non-GM maize from service to the end of lactation. GM maize-fed sows were heavier on day 56 of gestation (*P* = 0.04). Offspring from sows fed non-GM maize tended to be heavier at weaning (*P* = 0.08). Sows fed GM maize tended to have decreased serum total protein (*P* = 0.08), and increased serum creatinine (*P* = 0.09) and gamma glutamyl transferase activity (*P* = 0.07) on day 28 of lactation. Serum urea tended to be decreased on day 110 of gestation in GM maize-fed sows (*P* = 0.10) and in offspring at birth (*P* = 0.08). Sow haemogloblin concentration (*P* = 0.06), hematocrit (*P* = 0.06) and erythrocyte count (*P* = 0.01) were decreased in response to feeding GM maize. Both platelet count (*P* = 0.07) and mean cell haemogloblin concentration (MCHC; *P* = 0.05) were decreased on day 110 of gestation in GM maize-fed sows however, MCHC tended to be increased in offspring at birth (*P* = 0.08). There was minimal effect of feeding GM maize to sows during gestation and lactation on offspring serum biochemistry and haematology at birth and bodyweight at weaning.

**INTRODUCTION**

The genetic modification of crops has led to vast agronomic improvement and in turn rapid uptake of this relatively new technology. Since the introduction of genetically modified (GM) crops in 1996, the land area used for their cultivation has increased ninety-four fold reaching 160 million hectares worldwide in 2011. As the rate of adoption of GM crops continues to increase, so too will their consumption by animals reared for food. Consequently, the procurement of exclusively non-GM food ingredients will become increasing difficult and expensive.

Although numerous beneficial attributes such as resistance to insect damage, inclement growing conditions etc. are associated with the genetic modification of crops, the inclusion of GM crops in animal and human food has raised some concerns regarding their safety. Consumer concerns are mostly related to a perceived risk to health, development of toxicity, allergenicity of the transgenic proteins or the transfer of antibiotic resistance from the plant to bacteria residing in the gastrointestinal tract. Other concerns are associated with environmental issues, such as gene transfer from GM crops to indigenous plants, reducing biodiversity and the influence of the GM crops on non-target species.

GM crops are subject to rigorous pre-market risk assessment prior to authorization for release onto the European market. However, unintended consequences associated with the consumption of GM food by a genetically diverse population of humans and animals cannot adequately be evaluated during pre-market risk assessment. Post-market monitoring is required to ascertain if prolonged exposure to GM food results in responses that have been predicted by pre-market risk assessment or to reveal the presence of side effects that have been previously unpredicted.

Bt MON810 maize is engineered to express the truncated Cry1Ab toxin from *Bacillus thuringiensis*, which confers resistance to the European maize borer. This toxin interacts with the target larvae’s intestinal cells disrupting the intestinal lining leading to death. However, the toxin is believed to be non-toxic to mammals, birds, reptiles and amphibians due to a lack of specific receptors in the intestinal tract.

To date, there is a lack of long-term studies, conducted with a large number of animals over several generations with the objective of evaluating the effects of GM feeds on livestock species. Multigenerational studies evaluating the long-term effects of feeding GM feed have been confined to sheep, mice and rats and have not been conducted to date in pigs, perhaps the best recognised animal model for human physiology. Results from one multigenerational feeding study where mice were fed 33% NK603 × MON810 GM maize compared to non-GM maize concluded that litter size and weight in the 3^rd^ and 4^th^ litters of continuous breeding was decreased in the GM maize-fed group. However, concerns were raised by researchers as to the validity of this research due to perceived flaws in the statistical analysis of the data. The study was subsequently withdrawn by the Austrian government and the European Food Safety Authority (EFSA) Scientific Panel on Genetically Modified Organisms (GMO) concluded that “on the basis of the data presented the GMO Panel is of the opinion that no conclusions can be drawn from the report”.

The primary objective of this research was to examine the cross-generational effects of feeding Bt MON810 maize on maternal and offspring health in an animal more appropriately used as a model for humans. These investigations are part of a larger study examining the effects of long-term exposure of offspring from GM maize-fed sows to GM maize on growth and health indictors (Buzoianu *et al*., unpublished).

**MATERIALS AND METHODS**

**Animal Welfare**

The animal study complied with European Union Council Directive 91/630/EEC (outlines minimum standards for the protection of pigs) and 98/58/EC (concerns the protection of animals kept for farming purposes) and was approved by, and a license obtained from, the Irish Department of Health and Children (licence number B100/4147). Ethical approval was obtained from the Teagasc and Waterford Institute of Technology ethics committees.

**Experimental design and diets**

Twenty-four crossbred (Large White × Landrace) nulliparous sows and their offspring were used in a 20-week study. At service, sows (~ 165 kg) were blocked by BW and service date and randomly assigned to one of two dietary treatments: (1) non-GM isogenic parent line maize (Pioneer PR34N43) and (2) GM maize (Pioneer PR34N44 event MON810). Sows were fed dietary treatments from service throughout gestation and lactation until litters were weaned at day 28 post-farrowing (Table 1).

Seeds derived from GM Bt MON810 and non-GM parent line control maize (PR34N44 and PR34N43 varieties, respectively; Pioneer Hi-Bred, Sevilla, Spain) were grown simultaneously side by side in 2007 in Valtierra, Navarra, Spain by independent tillage farmers.independent The GM and non-GM control maize were purchased by the authors from the tillage farmers for use in this animal study*.* Samples from the GM and non-GM maize varieties were tested for chemical, amino acid and carbohydrate composition as well as for presence of the *cry1Ab* gene, pesticide contaminants, and mycotoxins as previously described by Walsh *et al.*.

All diets were manufactured and analysed for chemical composition and amino acid concentration as previously described by Walsh *et al.*. Sampling of the diets was conducted in accordance with international guidelines. All diets were formulated to meet or exceed the NRC requirements for pigs at the relevant stage of the production cycle. From selection at 100 kg until service, gilts were fed a non-GM gilt developer diet (13.67 MJ DE/kg, 6 g/kg lysine). Sows were fed either non-GM or GM maize based gestation diets from service until farrowing (~ 115 days). Following farrowing, sows were fed either non-GM or GM maize based lactation diets until litters were weaned (~ 28 days of age). Creep feed was not offered to suckling piglets.

**Housing and management**

Sows were purchased from Hermitage AI (Kilkenny, Ireland) as weanling pigs (~ 28 days old) and raised to ~165 kg in the Moorepark pig unit. During this time, the sows received diets that were free of GM ingredients. At ~ 100 kg, sows were selected for service based on weight and desirable conformation traits (leg conformation and nipple number and position) and housed in gilt accommodation until one week prior to service. Sows were restrictively fed during this time, receiving 2.5 kg of gilt developer diet per day. Synchronization of sow oestrus was achieved by administering 20 mg of altrenogest (Regumate, Intervet/Schering-Plough Animal Health, Bray, Ireland) per sow in feed for 18 days. Following administration of altrenogest, the sows were moved to the service area where they remained until service. A sexually mature boar was housed in the room to stimulate the sows to come into oestrus. Nine days prior to predicted oestrus, the sows were flush fed gilt developer diet (4 kg/day). Sows were inseminated with pooled semen from five Hylean MaxGro^TM^ boars (Hermitage AI, Kilkenny, Ireland) as soon as oestrus was detected and again 24 h later. After service, sows were transferred to dry sow accommodation and were penned individually in basket stalls (2.4 m × 0.6 m; O’Donovan Engineering, Coachford, Ireland) until day 110 of gestation. Environmental temperature was maintained between 20 and 22^o^C and sows had *ad libitum* access to water through a single nipple waterer per pen (Arato, Köln, Germany).

Verification of pregnancy was carried out 28 days after service using an ultrasound scanner (Oviscan 4 BCF Technology Ltd, Scotland, UK). Sows found not to be pregnant at the ultrasound scan or at any other time point (exhibiting heat or following abortion) were removed from the study. On day 74 and 99 of gestation sows were routinely vaccinated against *E. coli* (Porcoli Diluvac Forte; Intervet/Schering-Plough Animal Health). Deworming of sows was performed on day 110 of gestation by administering Zerofen 4% powder (Chanelle Animal Health Limited, Liverpool, UK) in feed (1 g/8 kg BW).

From day 110 of gestation until weaning, sows were accommodated in three farrowing rooms with 10 pens per room. National Pig Development company (NPD) type farrowing crates (O’Donovan Engineering) were used. Environmental temperature was maintained at 20^o^C except around farrowing when temperature was increased to 24^o^C for 48 h. Dietary treatments were equally represented in each room to avoid additional variation due to environment. Sows were fed 2 kg per day immediately post-farrowing and feed allowance was incrementally increased by 500 g per day until day 7 post-farrowing when sows were feeding *ad libitum.* Sows had access to feed from an *ad libitum* feeder (Daltec A/S, Tybovej 1, Egtved, Denmark) and unlimited access to water through a single nipple waterer (Arato, Köln, Germany) and supplemental water was provided by lever valve where necessary. Care was taken to minimize feed wastage and any soiled or stale feed was removed and accounted for. On day 114 of gestation, sows were treated with 2 mL of Enzaprost (5 mg/mL: CEVA Animal Health Ltd, UK) to induce farrowing 24-36 h later.

**Body weight and back-fat depth**

Back-fat depth and BW of sows were recorded at service, on day 56 and 110 of gestation and at weaning. Back-fat depth was recorded at the P2 position (65 mm down from the midline, at the level of the head of the last rib), using a Renco Lean Meater (Renco Corporation, Minneapolis, MN). The number of days post-weaning for sows to return to oestrus was also recorded (weaning to service interval).

**Variables measured at farrowing**

Individual BW of piglets in all litters was recorded at birth and weaning and average daily gain (ADG) was calculated during the suckling period. At farrowing, the fourth piglet born alive from each litter (n=12/treatment) was sacrificed by captive bolt stunning followed by exsanguination. Blood samples were taken for haematology and blood biochemical analysis as outlined below. The heart, kidneys, spleen and liver were removed, trimmed of any superficial fat or blood, blotted dry and weighed.

**Blood sampling and analysis**

Blood samples were collected from the external jugular vein of sows (n=12/treatment) prior to service, on day 28 and 110 of gestation and on day 28 of lactation for serum biochemical analysis and haematology*.* Blood samples for serum biochemistry analysis were collected in serum collection tubes (BD Vacutainer Systems, Franklin Lakes, NJ) and allowed to clot at room temperature for 2 to 3 h prior to centrifugation (1300 × g for 10 minutes)*.* Serum was collected and stored at -20^o^C for subsequent biochemical analysis*.* Serum samples were analysed for aspartate aminotransferase (AST), alanine aminotransferase (ALT), gamma glutamyltransferase (GGT), alkaline phosphatase (ALP), creatinine, urea and total protein (TP) as previously described by Buzoianu *et al*..

Whole blood samples were collected in K_2_EDTA blood collection tubes (Vacuette, Greiner Bio One Ltd, Gloucestershire, UK) and stored at room temperature prior to haematological analysis which was performed within six hours of collection*.* Whole blood samples were analysed using a Beckman Coulter Ac T Diff haematology analyser (Beckman Coulter Ltd., High Wycombe, UK)*.* The following parameters were determined; number of erythrocytes, haemoglobin concentration (Hgb), haematocrit (Hct), mean corpuscular volume (MCV), mean corpuscular haemoglobin (MCH), mean corpuscular haemoglobin concentration (MCHC), red cell distribution width (RDW), number of platelets (Plt) and mean platelet volume (MPV)*.* Samples were analysed according to the manufacturer’s instructions and general haematology recommendations.

**Statistical Analysis**

All data were analysed as a complete randomised block design using the GLM procedures of SAS (SAS Inst. Inc., Cary, NC) with pig used as the experimental unit*.* Treatment effect was tested against residual error terms with initial BW and service date as blocking factors*.* Sow BW, body composition, and offspring growth performance, blood biochemistry and haematology were analysed as a one-factor analysis of variance (ANOVA) using the GLM procedure of SAS*.* Organ weights were also analysed as a one-factor analysis of variance using the GLM procedure of SAS using birth weight as a covariate in the model*.* Sow serum biochemistry data and haematology were analysed as a repeated measure using the MIXED procedure of SAS with sampling day as the repeated variable. Simple main effects were obtained using the *slice* option in SAS. Means separation was performed using the Tukey-Kramer adjustment for multiple comparisons and values recorded prior to service were used as a covariate in the model*.* Fixed effects included pig, treatment and sampling day while block was included as a random effect in the model. For all tests, the level of significance was *P* < 0.05 and tendencies were reported for 0.05 < *P* < 0.10.

**RESULTS**

**Analysis of non-GM and GM diets**

Similar chemical and amino acid composition were observed between the non-GM and the GM diets (Table 1).

**Effect of feeding diets containing non-GM or GM maize on body weight, body composition of sows and growth rate of offspring**

Sows fed GM maize-based diets were on average 4 kg heavier on day 56 of gestation compared to sows fed non-GM maize diets (Table 2; *P* = 0.04). On day 110 of gestation, there was no BW difference between treatments. There was no difference in back-fat depth between treatments at any time during the study. The ADG of piglets or variation in ADG within litter during the suckling period was not affected by feeding GM maize to sows. Sows fed GM maize weaned 1.3 more piglets per litter than non-GM maize-fed sows; however, this difference was not statistically significant. Piglets from GM maize-fed sows tended to be 0.75 kg lighter at weaning than the piglets from non-GM maize-fed sows (*P* = 0.08). Litter weaning weight or the within litter variation in piglet weaning weight was not affected by feeding GM maize to sows. There was no difference in pre-weaning mortality levels per litter between treatments.

**Effect of feeding non-GM and GM maize diets to sows during gestation and lactation on serum biochemistry**

There tended to be a treatment × time interaction (*P* = 0.08) for total protein in serum (Table 3). On day 28 of lactation, total protein concentration in the serum of GM maize-fed sows tended to be lower than in sows fed non-GM maize diets. On day 110 of gestation, sows fed GM maize tended to have lower serum urea compared to non-GM maize-fed sows (*P* = 0.09) and serum urea was found to increase during gestation and lactation (*P* < 0.001). There tended to be a treatment × time interaction (*P* = 0.09) for serum creatinine. Serum creatinine was higher in GM maize-fed sows on day 28 of lactation compared to non-GM maize-fed sows. Serum creatinine increased during gestation but had decreased by day 28 of lactation (*P* < 0.001). There was no effect of treatment on ALT or ALP however, both parameters decreased over time (*P* < 0.001). On day 28 of lactation, GGT activity tended to be greater in the serum of GM maize-fed sows compared to non-GM maize-fed sows (*P* = 0.07). The concentration of GGT in serum decreased during gestation but serum concentrations were higher on day 28 of lactation than on day 28 of gestation (*P* < 0.001). There was no effect of treatment on serum AST during the study however, concentration of AST decreased during gestation and increased again during lactation (*P* = 0.002).

**Effect of feeding non-GM and GM maize diets to sows during gestation and lactation on sow haematology**

Sows fed GM maize during gestation and lactation had lower counts of erythrocytes in blood than sows fed non-GM maize (Table 4; *P* = 0.01). Erythrocyte count decreased for all sows during gestation and lactation (*P* < 0.001). Haemogloblin concentration (*P* = 0.06) and hematocrit (*P* = 0.06) also tended to be lower in the blood of GM maize-fed sows compared to non-GM maize fed sows. Both haemogloblin concentration and hematocrit also decreased over time in all sows (*P* < 0.001). There was no effect of treatment on MCV and MCH and MCV was found to increase over time (*P* < 0.001). On day 110 of gestation, feeding GM maize resulted in lower MCHC in blood (*P* = 0.05) compared to non-GM maize. The amount of MCHC in blood decreased during gestation and lactation (*P* < 0.001). There was no effect of feeding GM maize on RDW in blood and RDW was found to increase over time (*P* = 0.01). There tended to be a treatment × time interaction (*P* = 0.07) for the number of platelets in blood on day 110. On day 110 of gestation, there tended (*P* = 0.10) to be less platelets in the blood of GM maize-fed sows than non-GM maize-fed sows. There was no effect of treatment on MPV in blood and MPV was found to increase during gestation but decrease during lactation (*P* < 0.001).

**Effect of feeding non-GM and GM maize diets to sows during gestation on the serum biochemistry, haematology of newly born piglets and organ weight of offspring at birth**

There was no effect of feeding GM maize to sows during gestation on the heart, liver, kidneys and spleen weight of offspring at birth (Table 5). Total protein and creatinine in the serum of offspring at birth were unaffected by treatment however, the offspring of sows fed GM maize tended to have lower serum urea than offspring from sows fed non-GM maize (*P* = 0.08). The concentration of the liver enzymes ALT, AST, GGT and ALP in the serum of offspring at birth was not different between treatments. There was no effect of treatment on erythrocyte count, haemogloblin, hematocrit, MCV, MCH, RDW, platelet count and MPV measured in the blood of offspring at birth. The offspring of sows fed GM maize tended to have higher MCHC in blood than offspring from non-GM maize-fed sows (*P* = 0.08).

**DISCUSSION**

The research reported in this manuscript is the first study in pigs to examine the trans-generational effect of GM maize exposure on the health of both dam and offspring. Results from the current study indicates that feeding GM maize to sows during gestation does not affect body composition determined by back-fat depth and differences in BW observed between treatments at mid-gestation were not present in late gestation. Parallel to our findings Trabalza-Marinucci *et al*. found the feeding Bt176 maize to sheep over a three year period had no effect on BW or body condition score. A study where cows were fed Bt MON810 maize over two successive lactations revealed a decrease in body condition score and weight during the second lactation in the GM maize-fed group. Steinke *et al*. concluded that the observed differences in body condition score and weight observed in the cows could be attributed to biological variation and not GM maize exposure as values were still within the normal biological range for these animals.

Feeding GM maize to sows during gestation had no effect on offspring birth weight. However, GM maize-fed sows gave birth to numerically more live born piglets (2.5 extra piglets born/litter) than non-GM maize-fed sows and this may have lead to a significant effect on other parameters measured. This particular study was not designed to evaluate the effects of GM maize feeding on sow reproductive performance. Further appropriately designed studies are necessary to fully assess the effects of GM maize on the reproductive performance of sows. As a consequence of numerically more piglets born to and reared by GM maize-fed sows (1.3 extra piglets weaned/litter), the weaning weight of piglets from GM maize-fed sows was less than piglets from non-GM maize-fed sows. Piglets were not offered creep feed during the lactation period so any additional demand on the milk supply of the sow such as extra piglets would potentially have a negative impact on piglet growth rate. Similar to our findings, results from a sheep study found that feeding Bt176 maize over a three year period had no affect on fertility, twin rate, BW of lambs at birth, mortality or growth rate to weaning. The diet of lambs in this study was supplemented prior to weaning with a mixture of non-GM cereals and faba beans. Likewise, a study feeding Bt maize to rats found that birth rate and survival of offspring was not affected by treatment. However, Velimirov *et al*. found that mice fed 33% NK603 × MON810 GM maize during a multigenerational feeding study had decreased litter size and weight in the 3^rd^ and 4^th^ litters of continuous breeding compared to the non-GM maize-fed group. However, the statistical analysis of the data from this study was thought to be flawed and the EFSA Scientific Panel on GMO concluded that no conclusions can be drawn from the study Rhee *et al*. found that when rats were fed GM potato (herbicide resistant bar gene inserted) over successive generations, there was no effect on mating indices, fertility, gestation length or litter size and differences observed in fertility of the first generation were not attributed to diet.

We previously reported finding no effect of short-term exposure of Bt MON810 maize on serum total protein, creatinine, urea or liver enzymes AST, ALT, GGT and ALP in weanling pigs. Results from the present study found that feeding GM maize to sows during gestation and lactation resulted in increased serum creatinine and GGT activity and reduced serum total protein at the end of lactation and decreased serum urea at the end of gestation. However, the measured values were all within the normal reference range for pigs during these reproductive stages and all were trends towards statistical significance with the exception of serum creatinine which was significantly different. Likewise, offspring from GM maize-fed sows also had decreased serum urea concentrations at birth compared to offspring from non-GM maize-fed sows, however liver enzyme activity and liver weight at birth were not different between treatments. Liver dysfunction is characterised by increased GGT activity however, this must also be accompanied by a 10-70 fold increase in AST and 5-10 fold increase in ALP which was not observed in either sows or offspring in this study. Similar to our findings, at the end of lactation, ewes fed Bt176 maize also experienced an increase GGT activity in serum however, there was no effect on serum creatinine, total protein, urea, ALP or AST. In agreement with our results, serum total protein was found to be lower in rats fed Bt maize during a three generation study. Kilic and Akay also reported a decrease in plasma creatinine which was in contrast to our results. Likewise, Poulsen *et al.* observed lower creatinine concentration together with increased plasma activity of ALT in female rats fed GM rice. The change in sow serum urea documented in our study was transient and isolated to day 110 of gestation which questions the biological relevance of this change but this may account for the small decrease observed in serum urea in their offspring. Kidney dysfunction is characterised by elevated serum urea and creatinine, low serum TP and increased urinary protein to creatinine ratio*.* While urinary protein and creatinine were not measured during this study, serum total protein was reduced and serum creatinine was increased in sows fed GM-maize at the end of lactation in the present study. However, there was no change in serum urea and the observed changes in creatinine and total protein were small and remained within the normal reference range for these parameters in sows during gestation and lactation. Offspring serum total protein, creatinine and kidney weight were not different regardless of the dietary treatment of their dam. Therefore, there is lack of evidence to indicate that the changes in the blood biochemistry observed in sows during this study conform to a pattern indicative of either liver or kidney dysfunction or that these changes have any major effect on the organ function of offspring at birth.

Sows in the present study experienced a decline in erythrocyte count, haemoglobin and hematocrit concentrations during gestation and lactation. Haemoglobin concentration is known to decrease in sows during gestation and lactation, however, reductions in erythrocyte count and hematocrit have only been reported during gestation. In particular, sows fed GM maize during gestation and lactation experienced a greater reduction in erythrocyte count, haemoglobin concentration and hematocrit than non-GM maize fed sows. The iron requirement of sows increases during gestation however, iron deficiency is rare in sows as they have an ample reservoir of iron stored in body tissue. Gestational anaemia arising from iron deficiency is unlikely to be the cause of the decrease in erythrocyte count, haemoglobin concentration and hematocrit observed in GM maize-fed sows as these changes were not accompanied by a decrease in MCV which is indicative of anaemia. The volume of blood in which circulating metabolites are diluted increases during gestation and lactation and the magnitude of this increase is also associated with the number of developing foetus. In our study, GM maize-fed sows gave birth to on average 2.5 more piglets than non-GM maize-fed sows therefore, hemodilution was more pronounced in these sows and may account for the decrease in erythrocyte count, haemoglobin concentration and hematocrit observed following GM maize consumption. In support of this hypothesis, when litter size was included as a covariate in the statistical model used for analysis of these variables, the significant differences between treatments disappeared. Also, the observed differences in sow haematology were not passed on to the next generation as no treatment differences were detected in erythrocyte count, haemoglobin concentration and hematocrit in offspring. A study where haematological parameters were measured in response to feeding GM rice expressing Cry1Ab to rats found no affect of treatment. Trabalza-Marinucci *et al*. reported an increase in haemagloblin in sheep fed Bt176 maize however, the number of offspring born to the ewes was not different to the controls fed isogenic maize. The decrease in platelet count and MCHC observed at the end of gestation in the GM maize-fed sows in the present study was transient and minimal and is unlikely to be of biological significance. Offspring from sows fed GM-maize were found to have a tendency for an increase in MCHC at birth, however this was not accompanied by any other changes in haematology and was contrary to the decrease in MCHC found in their dams. Further research is ongoing by our research group to establish if changes observed in the offspring of GM maize-fed sows at birth will affect the lifetime performance of these pigs and this will help to determine the biological relevance of these changes.

In conclusion, feeding Bt MON810 maize to sows during gestation and lactation had no affect on BW or back-fat depth of the sow. Changes in sow blood biochemistry during the study in response to GM maize did not conform to a pattern indicative of organ dysfunction and were within the normal reference range for sows. Differences in the haematology of sows between treatments is likely to have been as a consequence of the increased litter size in GM maize-fed sows and appeared to be unrelated to GM maize exposure. There was minimal effect of feeding GM maize to sows during gestation and lactation on offspring serum biochemistry and haematology at birth and BW at weaning. However, further studies are currently underway to evaluate the lifetime health and growth performance of offspring from GM maize-fed sows which will further help to improve the safety assessments of GM organisms used for feed and food.

**Acknowledgements**

The research leading to these results was funded by the European Union's Seventh Framework Programme (FP7/2007-2013) under grant agreement n° 211820 and the Teagasc Walsh Fellowship programme and independently of any commercial input, financial or otherwise. None of the authors had a financial or personal conflict of interest in regard to the present study. P.G.L. and R.P.R. secured the funding for the research. P.G.L and G.E.G designed the experiment. M.C.W., S.G.B., P.G.L. and G.E.G. conducted the experiment. M.C.W., S.G.B., G.E.G. and O.O’D. conducted the laboratory analysis. M.C.W., S.G.B., and P.G.L. wrote the manuscript. All authors read and approved the final manuscript.

Table 1. Composition of sow gestation and lactation diets (as-as basis, %).

|  | Gestation | | Lactation | |
| --- | --- | --- | --- | --- |
| Ingredient, % | Non-GM | GM | Non-GM | GM |
| Maize (non-GM) | 86.55 | --- | 74.42 | --- |
| Maize (GM-MON810) | --- | 86.55 | --- | 74.42 |
| Soya Hi-Pro (non-GM) | 10.33 | 10.33 | 19.30 | 19.30 |
| Fat, soya oil | --- | --- | 3.02 | 3.02 |
| Lysine HCl (78.8) | 0.16 | 0.16 | 0.25 | 0.25 |
| DL-Methionine | --- | --- | 0.10 | 0.10 |
| L-Threonine (98) | --- | --- | 0.06 | 0.06 |
| Premix* | 0.10 | 0.10 | 0.10 | 0.10 |
| Salt feed grade | 0.40 | 0.40 | 0.40 | 0.40 |
| Dicalcium phosphate | 1.36 | 1.36 | 1.29 | 1.29 |
| Limestone flour | 1.10 | 1.10 | 1.06 | 1.06 |
| Analysed chemical composition (%) |  |  |  |  |
| Dry matter | 88.5 | 87.4 | 89.5 | 88.6 |
| Crude protein | 11.8 | 11.0 | 15.6 | 15.0 |
| Fat | 3.0 | 3.1 | 5.9 | 6.0 |
| Crude fibre | 1.6 | 1.5 | 1.6 | 1.9 |
| Ash | 4.0 | 4.0 | 4.6 | 4.3 |
| Lysine | 0.64 | 0.68 | 1.01 | 0.96 |
| Ca† | 7.6 | 7.6 | 7.5 | 7.5 |
| P† | 6.1 | 6.1 | 6.2 | 6.2 |
| Digestible energy, MJ of DE/kg‡ | 13.80 | 13.80 | 14.50 | 14.50 |

^*^Premix provided per kg of complete diet: Cu, 150 mg; Fe, 70 mg; Mn, 62 mg; Zn, 80 mg, I, 0.6 mg; Se, 0.2 mg; vitamin A, 10000 IU; vitamin D_3_, 1000 IU; vitamin E, 100 IU; vitamin K, 2 mg; vitamin B_12_, 15 μg; riboflavin, 5 mg; nicotinic acid, 12 mg; pantothenic acid, 10 mg; choline chloride, 500 mg; vitamin B_1_, 2 mg; and vitamin B_6_, 3 mg.

†Calculated values

‡Calculated from equation number 24 in Noblet and Perez using analysed values on an *as-is* basis.

Table 2. Effect of feeding Bt (MON810) maize to sows during gestation and lactation on sow body weight and back-fat depth and progeny growth performance.

(Mean values with their standard errors, *n* 12)

| Treatment |  | Non-GM maize | GM maize |  | SE |  | *P* |
| --- | --- | --- | --- | --- | --- | --- | --- |
| *Sow performance* |  |  |  |  |  |  |  |
| Body weight at service, kg |  | 164.3 | 165.2 |  | 0.40 |  | NS |
| Body weight at d 56 of gestation, kg |  | 193.8 | 197.8 |  | 1.20 |  | * |
| Body weight at d 110 of gestation, kg |  | 222.2 | 227.1 |  | 2.38 |  | NS |
| Back-fat depth at service, mm |  | 18.5 | 17.4 |  | 0.84 |  | NS |
| Back-fat depth at d 56 of gestation, mm |  | 18.9 | 18.7 |  | 0.80 |  | NS |
| Back-fat depth at d 110 of gestation, mm |  | 18.7 | 18.9 |  | 0.56 |  | NS |
| *Piglet growth performance during lactation* |  |  |  |  |  |  |  |
| Mean birth weight, kg*** |  | 1.45 | 1.33 |  |  |  |  |
| ADG, g/d |  | 229.7 | 206.4 |  | 10.12 |  | NS |
| Variation in ADG, % |  | 17.5 | 17.7 |  | 2.15 |  | NS |
| Number of piglets weaned/sow |  | 10.6 | 11.9 |  | 0.76 |  | NS |
| Mean weaning weight, kg |  | 7.65 | 6.90 |  | 0.286 |  | † |
| Litter weaning weight, kg |  | 79.8 | 81.2 |  | 6.49 |  | NS |
| Variation in weaning weight, % |  | 17.5 | 15.6 |  | 1.75 |  | NS |
| Pre-weaning mortality, number of pigs/litter |  | 1.05 | 1.42 |  | 0.248 |  | NS |

ADG, average daily gain.

Mean values were significantly different between two treatments: **P* < 0.05, †*P* < 0.10.

***No statistical analysis was conducted on piglet birth weight due to inadequate replication to detect statistical differences.

Table 3. Effect of feeding Bt (MON810) maize to sows during gestation and lactation on serum biochemistry.

(Mean values with their standard errors, *n* 12)

|  | Treatment | |  |  | *P* | | |
| --- | --- | --- | --- | --- | --- | --- | --- |
| Day | Non-GM maize | GM maize | Mean | SE | Treatment | Time | Treatment × Time |
| *Serum total protein, g/L* | | |  |  |  |  |  |
| d 28 of gestation | 75.0 | 74.0 | 74.5 | 1.65 | NS |  |  |
| d 110 of gestation | 73.5 | 74.8 | 74.2 | 1.62 | NS |  |  |
| d 28 of lactation | 75.2 | 71.9 | 73.5 | 1.62 | † |  |  |
| Mean | 74.6 | 73.6 |  | 1.41 | NS | NS | † |
| *Serum urea, mmol/L* | | |  |  |  |  |  |
| d 28 of gestation | 2.20 | 2.10 | 2.11 | 0.207 | NS |  |  |
| d 110 of gestation | 2.54 | 2.04 | 2.29 | 0.207 | † |  |  |
| d 28 of lactation | 4.23 | 4.26 | 4.29 | 0.216 | NS |  |  |
| Mean | 3.02 | 2.77 |  | 0.142 | NS | *** | NS |
| *Serum creatinine, µmol/L* | | |  |  |  |  |  |
| d 28 of gestation | 142.6 | 141.6 | 142.1 | 4.13 | NS |  |  |
| d 110 of gestation | 195.6 | 205.2 | 200.4 | 4.13 | NS |  |  |
| d 28 of lactation | 156.1 | 169.3 | 162.7 | 4.28 | * |  |  |
| Mean | 164.8 | 172.0 |  | 3.15 | NS | *** | † |
| *Alanine aminotransferase, units/L* | | |  |  |  |  |  |
| d 28 of gestation | 33.6 | 36.8 | 35.2 | 1.54 | NS |  |  |
| d 110 of gestation | 29.3 | 30.6 | 29.9 | 1.54 | NS |  |  |
| d 28 of lactation | 27.8 | 27.2 | 27.0 | 1.61 | NS |  |  |
| Mean | 29.9 | 31.5 |  | 1.04 | NS | *** | NS |
| *Alkaline phosphatase, units/L* | | |  |  |  |  |  |
| d 28 of gestation | 73.7 | 73.4 | 73.6 | 3.55 | NS |  |  |
| d 110 of gestation | 57.8 | 57.7 | 57.8 | 3.55 | NS |  |  |
| d 28 of lactation | 47.5 | 45.8 | 46.7 | 3.70 | NS |  |  |
| Mean | 59.7 | 59.0 |  | 2.38 | NS | *** | NS |
| *Gamma glutamyl transferase, units/L* | | |  |  |  |  |  |
| d 28 of gestation | 51.1 | 55.4 | 53.1 | 3.39 | NS |  |  |
| d 110 of gestation | 47.5 | 47.8 | 47.7 | 3.39 | NS |  |  |
| d 28 of lactation | 61.1 | 70.6 | 65.8 | 3.50 | † |  |  |
| Mean | 53.3 | 57.9 |  | 2.69 | NS | *** | NS |
| *Aspartate aminotransferase, units/L* | | |  |  |  |  |  |
| d 28 of gestation | 22.1 | 21.3 | 21.7 | 1.99 | NS |  |  |
| d 110 of gestation | 20.7 | 18.3 | 19.5 | 1.99 | NS |  |  |
| d 28 of lactation | 24.2 | 27.6 | 25.9 | 2.08 | NS |  |  |
| Mean | 22.3 | 22.4 |  | 1.47 | NS | ** | NS |

Mean values were significantly different between two treatments: **P* < 0.05, ***P* < 0.01, ****P* < 0.001, †*P* < 0.10.

Table 4. Effect of feeding Bt (MON810) maize to sows during gestation and lactation on haematology.

(Mean values with their standard errors, *n* 12)

|  | Treatment | |  |  | *P* | | |
| --- | --- | --- | --- | --- | --- | --- | --- |
| Day | Non-GM maize | GM maize | Mean | SE | Treatment | Time | Treatment × Time |
| *Erythrocyte, 1,000,000/μL* | | |  |  |  |  |  |
| d 28 of gestation | 7.99 | 7.29 | 7.64 | 0.096 | *** |  |  |
| d 110 of gestation | 6.90 | 6.30 | 6.61 | 0.089 | *** |  |  |
| d 28 of lactation | 5.99 | 5.36 | 5.68 | 0.089 | *** |  |  |
| Mean | 6.96 | 6.32 |  | 0.011 | *** | *** | NS |
| *Haemogloblin, g/dL* | | |  |  |  |  |  |
| d 28 of gestation | 15.6 | 15.0 | 15.3 | 0.21 | † |  |  |
| d 110 of gestation | 13.2 | 13.1 | 13.2 | 0.21 | NS |  |  |
| d 28 of lactation | 11.8 | 11.2 | 11.5 | 0.22 | † |  |  |
| Mean | 13.5 | 13.1 |  | 0.14 | † | *** | NS |
| *Hematocrit, L/L* | | |  |  |  |  |  |
| d 28 of gestation | 0.450 | 0.431 | 0.440 | 0.0091 | NS |  |  |
| d 110 of gestation | 0.393 | 0.380 | 0.386 | 0.0073 | NS |  |  |
| d 28 of lactation | 0.347 | 0.327 | 0.337 | 0.0073 | † |  |  |
| Mean | 0.397 | 0.379 |  | 0.0040 | † | *** | NS |
| *MCV, fL* | | |  |  |  |  |  |
| d 28 of gestation | 56.5 | 57.2 | 56.8 | 0.45 | NS |  |  |
| d 110 of gestation | 57.8 | 58.1 | 57.9 | 0.45 | NS |  |  |
| d 28 of lactation | 59.0 | 59.0 | 59.0 | 0.47 | NS |  |  |
| Mean | 57.7 | 58.1 |  | 0.34 | NS | *** | NS |
| *MCH, g/dL* | | |  |  |  |  |  |
| d 28 of gestation | 19.6 | 19.9 | 19.8 | 0.31 | NS |  |  |
| d 110 of gestation | 20.2 | 20.0 | 20.1 | 0.28 | NS |  |  |
| d 28 of lactation | 20.3 | 20.0 | 20.1 | 0.28 | NS |  |  |
| Mean | 20.1 | 20.0 |  | 0.25 | NS | NS | NS |
| *MCHC, %* | | |  |  |  |  |  |
| d 28 of gestation | 34.4 | 34.3 | 34.4 | 0.16 | NS |  |  |
| d 110 of gestation | 34.6 | 34.1 | 34.3 | 0.13 | * |  |  |
| d 28 of lactation | 33.8 | 33.8 | 33.8 | 0.13 | NS |  |  |
| Mean | 34.3 | 34.1 |  | 0.09 | NS | *** | NS |
| *RDW, %* | | |  |  |  |  |  |
| d 28 of gestation | 18.0 | 17.7 | 17.8 | 0.25 | NS |  |  |
| d 110 of gestation | 17.6 | 17.8 | 17.7 | 0.25 | NS |  |  |
| d 28 of lactation | 18.2 | 18.3 | 18.3 | 0.26 | NS |  |  |
| Mean | 17.9 | 17.9 |  | 0.20 | NS | ** | NS |
| *Platelets, 1000/μL* | | |  |  |  |  |  |
| d 28 of gestation | 252.6 | 269.6 | 261.1 | 24.0 | NS |  |  |
| d 110 of gestation | 264.1 | 215.8 | 239.9 | 23.1 | NS |  |  |
| d 28 of lactation | 348.3 | 383.0 | 365.7 | 24.0 | NS |  |  |
| Mean | 288.3 | 289.5 |  | 17.9 | NS | NS | NS |
| *MPV, fL* | | |  |  |  |  |  |
| d 28 of gestation | 7.99 | 8.17 | 8.08 | 0.15 | NS |  |  |
| d 110 of gestation | 9.12 | 8.93 | 9.02 | 0.15 | NS |  |  |
| d 28 of lactation | 8.47 | 8.66 | 8.56 | 0.16 | NS |  |  |
| Mean | 8.52 | 8.59 |  | 0.10 | NS | *** | NS |

MCV, mean corpuscular volume; MCH, mean corpuscular haemoglobin; MCHC, mean corpuscular haemoglobin

concentration; RDW, red cell distribution width; MPV, mean platelet volume.

Mean values were significantly different between two treatments: **P* < 0.05, ***P* < 0.01, ****P* < 0.001, †*P* < 0.10.

Table 5. Effect of feeding Bt (MON810) maize to sows during gestation on progeny organ weight, blood biochemistry and haematology at birth.

(Mean values with their standard errors, *n* 12)

| Treatment |  | Non-GM maize | GM maize |  | SE |  | *P* |
| --- | --- | --- | --- | --- | --- | --- | --- |
| *Organ weight, g** |  |  |  |  |  |  |  |
| Heart |  | 9.85 | 9.84 |  | 0.340 |  | NS |
| Kidneys |  | 9.88 | 9.77 |  | 0.539 |  | NS |
| Spleen |  | 1.28 | 1.16 |  | 0.069 |  | NS |
| Liver |  | 39.7 | 36.9 |  | 1.93 |  | NS |
| Serum total protein |  | 22.2 | 22.8 |  | 0.79 |  | NS |
| Serum urea |  | 3.08 | 2.41 |  | 0.249 |  | † |
| Serum creatinine |  | 161.3 | 156.3 |  | 12.52 |  | NS |
| *Liver enzymes, units/L* |  |  |  |  |  |  |  |
| Alanine aminotransferase |  | 11.1 | 13.0 |  | 1.04 |  | NS |
| Aspartate aminotransferase |  | 60.9 | 70.8 |  | 18.28 |  | NS |
| Gamma glutamyl transferase |  | 55.2 | 64.3 |  | 4.77 |  | NS |
| Alkaline phosphatase |  | 1968.6 | 2087.7 |  | 306.59 |  | NS |
| *Haematology* |  |  |  |  |  |  |  |
| Erythrocyte, 1,000,000/μL |  | 5.79 | 4.50 |  | 0.707 |  | NS |
| Haemogloblin, g/dL |  | 10.92 | 8.05 |  | 1.904 |  | NS |
| Hematocrit, L/L |  | 1.73 | 5.33 |  | 2.56 |  | NS |
| MCV, fL |  | 81.5 | 62.5 |  | 14.49 |  | NS |
| MCH, g/dL |  | 19.13 | 22.04 |  | 1.346 |  | NS |
| MCHC, % |  | 29.54 | 30.50 |  | 0.355 |  | † |
| RDW, % |  | 18.44 | 18.31 |  | 0.453 |  | NS |
| Platelets, 1000/μL |  | 154.2 | 117.4 |  | 22.45 |  | NS |
| MPV, fL |  | 9.65 | 9.56 |  | 0.361 |  | NS |

MCV, mean corpuscular volume; MCH, mean corpuscular haemoglobin; MCHC, mean corpuscular haemoglobin concentration; RDW, red cell distribution width; MPV, mean platelet volume.

*Organ weight data were analysed using birth weight as a covariate in the model.

Mean values were significantly different between two treatments: †*P* < 0.10.
