# Supplementary material for: Effects of Feeding Bt MON810 Maize to Pigs for 110 Days on Peripheral Immune Response and Digestive Fate of the cry1Ab Gene and Truncated Bt Toxin
Source: PLoS One. 2012 May 4;7(5):e36141. doi: 10.1371/journal.pone.0036141 (PMC3345032; doi:10.1371/journal.pone.0036141)
Supplement: Table S1 — Effects of feeding of Bt and isogenic maize on haematological parameters in growing pigs. (DOC) [file pone.0036141.s002.doc]

**Table S1**. Effects of feeding of Bt and isogenic maize on haematological parameters in growing pigs. All values are shown ± SE.

|  |  | Treatment | | | |  |  | *P* - value | | |
| --- | --- | --- | --- | --- | --- | --- | --- | --- | --- | --- |
| Day |  | Isogenic^1^ | Bt^2^ | Isogenic/Bt^3^ | Bt/isogenic^4^ | Mean |  | Treatment | Time | Treatment × Time |
|  |  | Erythrocyte, 1,000,000/μL | | | |  |  |  |  |  |
| 30 |  | 6.5 ± 0.21 | 6.8 ± 0.18 | 6.5 ± 0.20 | 7.1 ± 0.23 | 6.7 ± 0.09 |  | 0.17 |  |  |
| 60 |  | 7.3 ± 0.22 | 7.2 ± 0.20 | 7.2 ± 0.21 | 7.7 ± 0.23 | 7.4 ± 0.10 |  | 0.42 |  |  |
| 100 |  | 7.2^xy^ ± 0.20 | 6.8^y^ ± 0.19 | 6.8^y^ ± 0.21 | 7.7^x^ ± 0.23 | 7.1 ± 0.09 |  | 0.02 |  |  |
| Mean |  | 7.0 ± 0.16 | 6.9 ± 0.14 | 6.8 ± 0.16 | 7.5 ± 0.20 |  |  | 0.11 | 0.0001 | 0.43 |
|  |  | Haemogloblin, g/dL | | | |  |  |  |  |  |
| 30 |  | 11.2 ± 0.55 | 11.8 ± 0.47 | 11.5 ± 0.49 | 12.0 ± 0.64 | 11.6 ± 0.22 |  | 0.79 |  |  |
| 60 |  | 13.1 ± 0.54 | 12.8 ± 0.50 | 12.8 ± 0.52 | 13.4 ± 0.64 | 13.0 ± 0.24 |  | 0.85 |  |  |
| 100 |  | 12.9 ± 0.53 | 12.4 ± 0.48 | 12.0 ± 0.52 | 13.5 ± 0.64 | 12.7 ± 0.23 |  | 0.28 |  |  |
| Mean |  | 12.4 ± 0.47 | 12.3 ± 0.40 | 12.1 ± 0.43 | 13.0 ± 0.59 |  |  | 0.64 | < 0.0001 | 0.48 |
|  |  | Hematocrit, L/L | | | |  |  |  |  |  |
| 30 |  | 0.36 ± 0.02 | 0.39 ± 0.01 | 0.40 ± 0.02 | 0.40 ± 0.02 | 0.38 ± 0.01 |  | 0.40 |  |  |
| 60 |  | 0.40 ± 0.02 | 0.41 ± 0.02 | 0.38 ± 0.02 | 0.42 ± 0.02 | 0.40 ± 0.01 |  | 0.95 |  |  |
| 100 |  | 0.38 ± 0.02 | 0.38 ± 0.01 | 0.40 ± 0.02 | 0.41 ± 0.02 | 0.39 ± 0.01 |  | 0.61 |  |  |
| Mean |  | 0.38 ± 0.01 | 0.39 ± 0.01 | 0.38 ± 0.01 | 0.4± 0.02 |  |  | 0.65 | 0.005 | 0.75 |
|  |  | MCV^5^, fL | | | |  |  |  |  |  |
| 30 |  | 54.5 ± 1.48 | 57.1 ± 1.27 | 55.8 ± 1.47 | 56.4 ± 1.81 | 56.0 ± 0.67 |  | 0.71 |  |  |
| 60 |  | 53.9 ± 1.46 | 55.9 ± 1.31 | 56.3 ± 1.52 | 55.6 ± 1.77 | 55.4 ± 0.69 |  | 0.76 |  |  |
| 100 |  | 52.5 ± 1.45 | 54.8 ± 1.28 | 55.1 ± 1.54 | 54.2 ± 1.77 | 54.2 ± 0.68 |  | 0.68 |  |  |
| Mean |  | 53.7 ± 1.39 | 55.9 ± 1.19 | 55.7 ± 1.41 | 55.4 ± 1.71 |  |  | 0.73 | 0.001 | 0.87 |
|  |  | MCH^6^, g/dL | | | |  |  |  |  |  |
| 30 |  | 16.9 ± 0.51 | 17.3 ± 0.44 | 17.6 ± 0.47 | 17.6 ± 0.54 | 17.3 ± 0.21 |  | 0.83 |  |  |
| 60 |  | 17.4 ± 0.51 | 17.5 ± 0.46 | 17.7 ± 0.49 | 18.1 ± 0.54 | 17.7 ± 0.23 |  | 0.84 |  |  |
| 100 |  | 17.7 ± 0.50 | 17.9 ± 0.45 | 17.7 ± 0.49 | 18.1 ± 0.54 | 17.8 ± 0.22 |  | 0.93 |  |  |
| Mean |  | 17.3 ± 0.47 | 17.6 ± 0.41 | 17.6 ± 0.44 | 17.9 ± 0.50 |  |  | 0.91 | 0.01 | 0.81 |
|  |  | MCHC^7^, % | | | |  |  |  |  |  |
| 30 |  | 31.2 ± 0.51 | 30.4 ± 0.44 | 31.9 ± 0.51 | 31.2 ± 0.61 | 31.2 ± 0.24 |  | 0.23 |  |  |
| 60 |  | 32.7 ± 0.52 | 31.4 ± 0.47 | 31.7 ± 0.52 | 32.1 ± 0.58 | 32.0 ± 0.25 |  | 0.38 |  |  |
| 100 |  | 33.9 ± 0.49 | 32.8 ± 0.46 | 32.4 ± 0.56 | 33.0 ± 0.58 | 33.0 ± 0.24 |  | 0.38 |  |  |
| Mean |  | 32.6 ± 0.44 | 31.6 ± 0.39 | 32.0 ± 0.46 | 32.1 ± 0.52 |  |  | 0.49 | 0.0001 | 0.49 |
|  |  | RDW^8^, % | | | |  |  |  |  |  |
| 30 |  | 20.4 ± 1.35 | 17.1 ± 1.11 | 19.8 ± 1.18 | 20.3 ± 1.59 | 19.4 ± 0.53 |  | 0.24 |  |  |
| 60 |  | 21.2 ± 1.34 | 17.6 ± 1.15 | 19.7 ± 1.22 | 19.5 ± 1.59 | 19.5 ± 0.56 |  | 0.23 |  |  |
| 100 |  | 21.6 ± 1.33 | 17.9 ± 1.13 | 21.7 ± 1.22 | 19.8 ± 1.59 | 20.2 ± 0.54 |  | 0.13 |  |  |
| Mean |  | 21.1 ± 1.27 | 17.5 ± 1.04 | 20.4 ± 1.11 | 19.9 ± 1.53 |  |  | 0.21 | 0.07 | 0.36 |
|  |  | Platelets, 1000/μL | | | |  |  |  |  |  |
| 30 |  | 511.4 ± 67.82 | 572.1 ± 58.82 | 637.0 ± 70.29 | 664.9 ± 84.06 | 596.3 ± 28.76 |  | 0.49 |  |  |
| 60 |  | 454.1 ± 65.60 | 547.1 ± 63.61 | 518.4 ± 77.37 | 545.5 ± 84.06 | 516.3 ± 31.54 |  | 0.80 |  |  |
| 100 |  | 467.1 ± 63.77 | 465.4 ± 60.82 | 435.6 ± 77.37 | 476.5 ± 84.06 | 461.2 ± 30.05 |  | 0.98 |  |  |
| Mean |  | 477.5 ± 55.91 | 528.2 ± 49.19 | 530.3 ± 65.08 | 562.3 ± 75.04 |  |  | 0.86 | <0.0001 | 0.54 |
|  |  | MPV^9^, fL | | | |  |  |  |  |  |
| 30 |  | 8.6 ± 0.70 | 9.4 ± 0.60 | 9.9 ± 0.63 | 10.0 ± 0.89 | 9.5 ± 0.30 |  | 0.62 |  |  |
| 60 |  | 9.0 ± 0.69 | 10.0 ± 0.63 | 9.5 ± 0.65 | 9.5 ± 0.89 | 9.5 ± 0.32 |  | 0.81 |  |  |
| 100 |  | 7.9 ± 0.68 | 8.7 ± 0.61 | 8.2 ± 0.65 | 8.9 ± 0.89 | 8.4 ± 0.31 |  | 0.80 |  |  |
| Mean |  | 8.5 ± 0.64 | 9.4 ± 0.55 | 9.2 ± 0.58 | 9.5 ± 0.85 |  |  | 0.82 | < 0.0001 | 0.46 |

^1^Isogenic: isogenic maize diet for 110 days.

^2^Bt: Bt maize diet for 110 days.

^3^Isogenic/Bt: isogenic maize diet for 30 days followed by Bt maize diet for 80 days.

^4^Bt/isogenic: Bt maize diet for 30 days followed by isogenic maize diet for 80 days.

^5^MCV; mean corpuscular volume.

^6^MCH; mean corpuscular haemoglobin.

^7^MCHC; mean corpuscular haemoglobin concentration.

^8^RDW; red cell distribution width.

^9^MPV; mean platelet volume.

^xy^Within a row means without a common superscript differ by *P* < 0.05 by means separation using Tukey-Kramer adjustment for multiple comparisons.
